# Myeloid Ikaros–SIRT1 signaling axis regulates hepatic inflammation and pyroptosis in ischemia-stressed mouse and human liver

**DOI:** 10.1016/j.jhep.2021.11.026

**Published:** 2021-12-03

**Authors:** Kentaro Kadono, Shoichi Kageyama, Kojiro Nakamura, Hirofumi Hirao, Takahiro Ito, Hidenobu Kojima, Kenneth J. Dery, Xiaoling Li, Jerzy W. Kupiec-Weglinski

**Affiliations:** 1 Dumont-UCLA Transplantation Center, Department of Surgery, Division of Liver and Pancreas Transplantation, David Geffen School of Medicine at UCLA, Los Angeles, CA 90095, USA;; 2 Division of Hepato-Biliary-Pancreatic Surgery and Transplantation, Department of Surgery, Graduate School of Medicine, Kyoto University, Kyoto, Japan;; 3 Signal Transduction Laboratory, National Institute of Environmental Health Sciences (NIEHS), Research Triangle Park, NC 27709, USA

**Keywords:** Ikaros, SIRT1, pyroptosis, liver inflammation, liver transplantation, macrophages

## Abstract

**Background & Aims:**

Although Ikaros (IKZF1) is a well-established transcriptional regulator in leukocyte lymphopoiesis and differentiation, its role in myeloid innate immune responses remains unclear. Sirtuin 1 (SIRT1) is a histone/protein deacetylase involved in cellular senescence, inflammation, and stress resistance. Whether SIRT1 signaling is essential in myeloid cell activation remains uncertain, while the molecular communication between Ikaros and SIRT1, two major transcriptional regulators, has not been studied.

**Methods:**

We undertook molecular and functional studies to interrogate the significance of the myeloid Ikaros–SIRT1 axis in innate immune activation and whether it may serve as a homeostatic sentinel in human liver transplant recipients (hepatic biopsies) and murine models of sterile hepatic inflammation (liver warm ischemia-reperfusion injury in wild-type, myeloid-specific *Sirt1*-knockout, and CD11b-DTR mice) as well as primary bone marrow-derived macrophage (BMM) cultures (Ikaros silencing *vs*. overexpression).

**Results:**

In our clinical study, we identified increased post-reperfusion hepatic Ikaros levels, accompanied by augmented inflammasome signaling yet depressed SIRT1, as a mechanism of hepatocellular damage in liver transplant recipients. In our experimental studies, we identified infiltrating macrophages as the major source of Ikaros in IR-stressed mouse livers. Then, we demonstrated that Ikaros-regulated pyroptosis – induced by canonical inflammasome signaling in BMM cultures – was SIRT1 dependent. Consistent with the latter, myeloid-specific Ikaros signaling augmented hepatic pyroptosis to aggravate pro-inflammatory responses *in vivo* by negatively regulating SIRT1 in an AMPK-dependent manner. Finally, myeloid-specific SIRT1 was required to suppress pyroptosis, pro-inflammatory phenotype, and ultimately mitigate hepatocellular injury in ischemia-stressed murine livers.

**Conclusion:**

These findings identify the Ikaros–SIRT1 axis as a novel mechanistic biomarker of pyroptosis and a putative checkpoint regulator of homeostasis in response to acute hepatic stress/injury in mouse and human livers.

## Lay summary

This report describes how crosstalk between Ikaros and SIRT1, two major transcriptional regulators, influence acute hepatic inflammation in murine models of liver ischemia-reperfusion injury and liver transplant recipients. We show that the myeloid Ikaros–SIRT1 axis regulates inflammasome-pyroptotic cell death and hepatocellular damage in stressed livers. Thus, the Ikaros-SIRT1 axis may serve as a novel checkpoint regulator that is required for homeostasis in response to acute liver injury in mice and humans.

## Graphical abstract

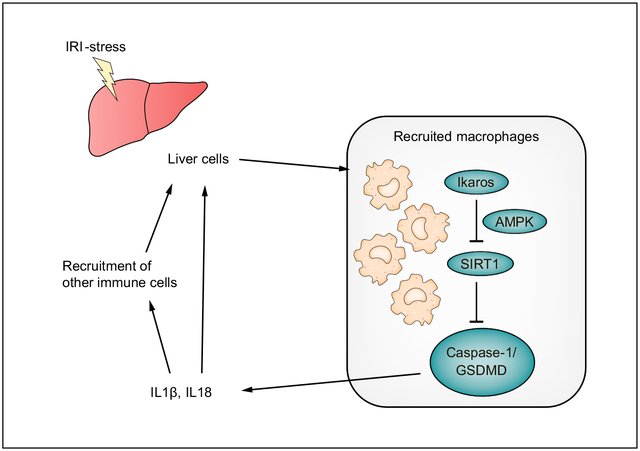

## Introduction

Orthotopic liver transplantation (OLT) has become the standard of care for end-stage liver disease and hepatic malignancies.^1^ By leading to early allograft dysfunction and failure, hepatic ischemia-reperfusion injury (IRI) represents a risk factor for acute and chronic OLT rejection and contributes to the shortage of donor organs.^2^ Despite compromising clinical outcomes, the mechanisms accounting for liver IRI are not well understood.

Liver IRI represents an innate immune continuum of pro-inflammatory cytokine release and hepatocyte death. Our group was one of the first to document that macrophage Toll-like receptor 4 (TLR4) signaling triggers a sterile hepatic inflammatory cascade in OLT recipients.^3^ TLR4-mediated canonical activation of the inflammasome complex was associated with a more recently identified inflammatory cell death termed pyroptosis.^4^ The inflammasome cleaves caspase-1, resulting in proteolytic cleavage of Gasdermin D (GSDMD), the executor of pyroptosis needed for IL1β and IL18 secretion.^5^ Its N-terminal domain (GSDMD-N) then translocates to the plasma membrane, causing water influx, cell swelling/lysis, and release of mature IL1β/IL18, which exacerbates immune cell recruitment.^6^ We have reported on the TLR-primed inflammasome as a critical signaling platform that detects stressors (*e.g*., damage-associated molecular patterns) in hepatic IRI^7,8^ while others targeted GSDMD to focus on pyroptosis in innate immune cell^9^ and Kupffer cell^10,11^ death. However, little is known about how recruited macrophages might trigger pyroptosis in acute liver inflammation.

Ikaros family zinc finger protein 1 (IKZF1/Ikaros), a conserved nuclear transcriptional factor well-studied in hemato-lymphoid lineage development/differentiation,^12^ serves as a repressor of inflammatory genes^13^ and regulator of the inflammatory response in mature T cells.^14^ By controlling self-antigen mediated B–T cell interactions and restraining TLR signaling, Ikaros acts as a “guardian” preventing autoimmunity/promoting lymphocyte homeostasis.^15^ Besides its role in macrophage NF-κB chromatin binding and gene expression in response to lipopolysaccharide (LPS),^16^ the function of Ikaros in myeloid cell innate immunity remains elusive.

Sirtuin 1 (SIRT1; silent mating type information regulation 2 homolog 1), a member of the class III histone/protein deacetylases, is involved in cellular senescence, inflammation, and stress resistance. While macrophage SIRT1-deficiency triggers NF-κB activation and pro-inflammatory gene programs,^17^ we have reported that pharmacologic SIRT1 overexpression promotes an anti-inflammatory phenotype and attenuates sterile inflammation in IR-stressed mouse livers.^18,19^ Consistent with SIRT1 orchestrating macrophage phenotypes in the formation of murine abdominal aortic aneurysms,^20^ we previously identified that SIRT1-driven M2-type macrophage polarization promoted hepatic rejuvenation in mouse and human OLT.^18,21^ However, whether SIRT1 regulates myeloid cell inflammasome activation and pyroptosis to promote the anti-inflammatory phenotype in IR-stressed livers remains unknown. Although Ikaros signaling may control the AMP-activated protein kinase (AMPK) metabolic pathway^22,23^ through SIRT1 and AMPK regulation,^24^ the molecular communication between Ikaros and SIRT1, two major transcriptional gene regulators, has not been studied.

To interrogate the significance of the Ikaros–SIRT1 myeloid axis in the activation of hepatic innate immunity induced by IR stress, we conducted molecular/functional studies encompassing primary mouse macrophage cultures, mouse models of sterile hepatic inflammation, and human OLT recipients.

## Patients and methods

### Clinical liver transplant study

The study was approved by the UCLA Institutional Research Board (IRB #13–000143). Patients provided informed consent before they participated in the study. As specified by UCLA protocols, we performed a retrospective analysis of 55 adult patients who underwent OLT (May 2013-August 2015) and received routine standard of care and immunosuppressive therapy. Recipients who underwent re-transplantation were excluded from the study. Donor livers, procured from donation after brain death or cardiac death, were perfused and stored in UW solution (Niaspan; Bristol-Meyers Squibb, Princeton, NJ). Protocol Tru-Cut needle biopsies were obtained from the left liver lobe about 2 h after portal reperfusion (before abdominal closure) and snap-frozen. Hepatic biopsies were screened by quantitative reverse-transcription PCR (qRT-PCR) with *GAPDH* normalization for *IKZF1, TLR4*, Cathepsin G, *CD80, CD86, CXCL10*, and western blots with β-actin normalization for SIRT1 and cleaved caspase-1 expression. Recipient blood samples were evaluated for serum alanine aminotransferase (sALT)/serum aspartate aminotransferase (sAST) levels.

### Animals

The study was approved by the UCLA Animal Research Committee (ARC #1999–094). C57BL/6 male mice: wild-type (WT), CD11b-DTR (Jackson Laboratory, Bar Harbor, ME), FLOX and myeloid-specific SIRT1-deficient (m*Sirt1*-KO) (NIEHS, Research Triangle Park, NC) were used. Animals were housed under pathogen-free conditions and received human care outlined in the Guide for Care and Use of Laboratory Animals (National Academies Press, 2011).

### Liver IRI mouse models

Mice were anesthetized, injected with heparin (100 U/kg), and an atraumatic clip was used to interrupt the hepatic artery/portal venous blood supply to the left/middle liver lobes.^25^ After 60 min of partial (75%) warm ischemia, the clamp was removed, and mice were sacrificed at 6 h of reperfusion. The sALT levels were measured with Infinity ALT Liquid Stable Reagent (Thermo Scientific, Rockford, IL).

To focus on macrophage-specific Ikaros function, CD11b-DTR mice were first treated with diphtheria toxin (DT; 25 ng/g i.v. at day −1) to deplete native CD11b+ cells,^26^ and then infused with *in vitro* generated siRNA-Ikaros *vs*. siRNA-Control macrophages (5×10^5^ cells i.v.) at 1 h before hepatic IR-insult. DT treatment does not trigger hepatotoxicity in WT mice.^26^

For further details regarding the materials and methods used, please refer to the CTAT table and supplementary information.

## Results

### The pro-inflammatory function of Ikaros in human liver transplantation

**Fig. 1.**
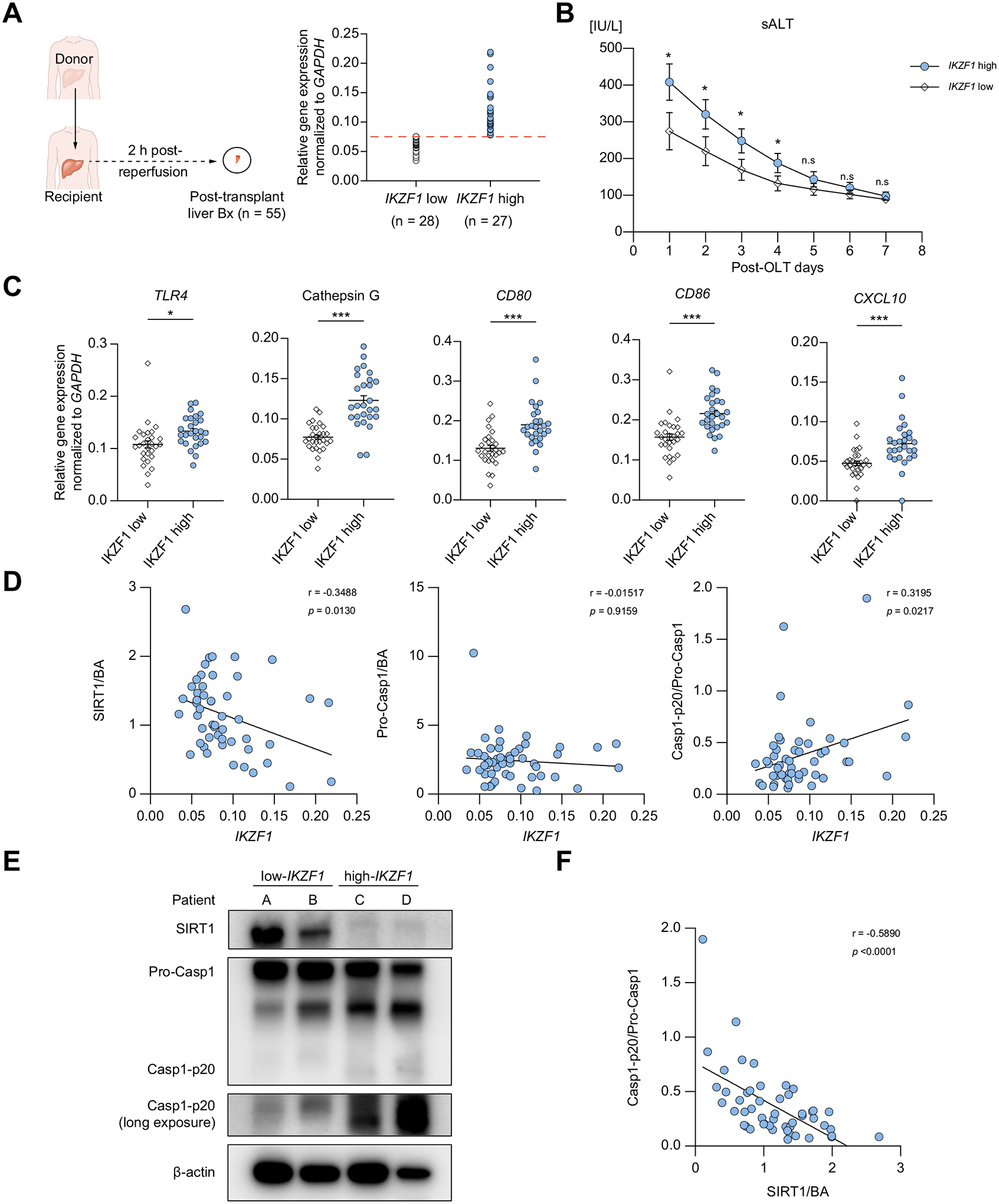
High *IKZF1* gene levels correlate with enhanced inflammation/hepatocellular damage but low SIRT1 expression/high cleaved caspase-1 levels in human OLT. (A) Hepatic biopsies (Bx) were collected at 2 h after reperfusion, followed by qRT-PCR screening for *IKZF1, TLR4*, Cathepsin G, *CD80, CD86, CXCL10* with *GAPDH* normalization and western blot evaluation for SIRT1 and cleaved caspase-1 (p20) with β-actin normalization. Fifty-five human OLT cases were classified into low (n = 28) and high (n = 27) *IKZF1* gene expression groups. (B) sALT levels in OLT recipients. Square points: IKZF1-low; dot points: IKZF1-high group. Data are shown as mean ± SEM. (C) qRT-PCR-assisted detection of mRNA coding for *TLR4*, Cathepsin G, *CD80, CD86, CD68, CXCL10*, normalized to *GAPDH*. Data are shown in a dot plot and bars indicative of mean ± SEM. Statistical analyses with 2-tailed Mann-Whitney *U* test. *p <0.05; **p <0.01; ***p <0.001. (D) Correlation between IKZF1 and SIRT1, pro-Casp1/Casp1-p20 ratio, analyzed by non-parametric Spearman`s method. (n = 51) (E) Representative western blots from *IKZF*-low and *IKZF*-high liver Bx with β-actin normalization. (F) Relationship between SIRT1/Casp1-p20 ratio. Correlations were analyzed by non-parametric Spearman`s method. Bx, biopsy; OLT, orthotopic liver transplantation; qRT-PCR, quantitative reverse-transcription PCR; sALT, serum alanine aminotransferase.

We aimed first to retrospectively evaluate Ikaros expression and its correlation with hepatocellular function in liver transplant recipients. Fifty-five human OLT biopsies, collected about 2 h after portal reperfusion, were classified into low (n = 28) *vs*. high (n = 27) *IKZF1* gene expression groups (Fig. 1A). As shown in Table S1, the *IKZF1*-high expression cohort had a significantly higher BMI (*p* = 0.0441). At the same time, there was no correlation between *IKZF1* levels and recipient sex, race, height, ABO compatibility, or preoperative serum AST/ALT levels. In addition, we found no correlation between *IKZF1* grouping and donor/graft variables, including sex, height, BMI, or warm/cold graft ischemia times (Table S2). Patients with low hepatic *IKZF1* expression showed improved early OLT function, evidenced by lower sALT (*p* <0.05) compared to the *IKZF1*-high expression group (Fig. 1B). The latter cases were characterized by increased hepatic TLR4 (innate activation marker; *p* = 0.0010), Cathepsin G (neutrophil marker; *p* <0.0001), and pro-inflammatory profile (*CD86, CD80, CXCL10*; *p* <0.0001) (Fig. 1C).

Notably, the expression of the SIRT1 protein in human OLT correlated negatively with *IKZF1* (r = −0.3488, *p* = 0.0130), while cleaved caspase-1 p-20 (Casp1-p20) correlated positively with *IKZF1* (r = 0.3195, *p* = 0.0217) (Fig. 1D,E) levels. At the same time, SIRT1 expression correlated negatively with Casp1-p20 (r = −0.5890 *p* <0.0001) (Fig. 1F). These results suggest a functional significance for the Ikaros–SIRT1 axis in IR-stressed human livers and identify increased post-reperfusion Ikaros levels, accompanied by enhanced inflammasome signaling, as a mechanism for hepatocellular damage in OLT recipients.

### Ikaros expression by recruited macrophages in IR-stressed WT mouse liver

**Fig. 2.**
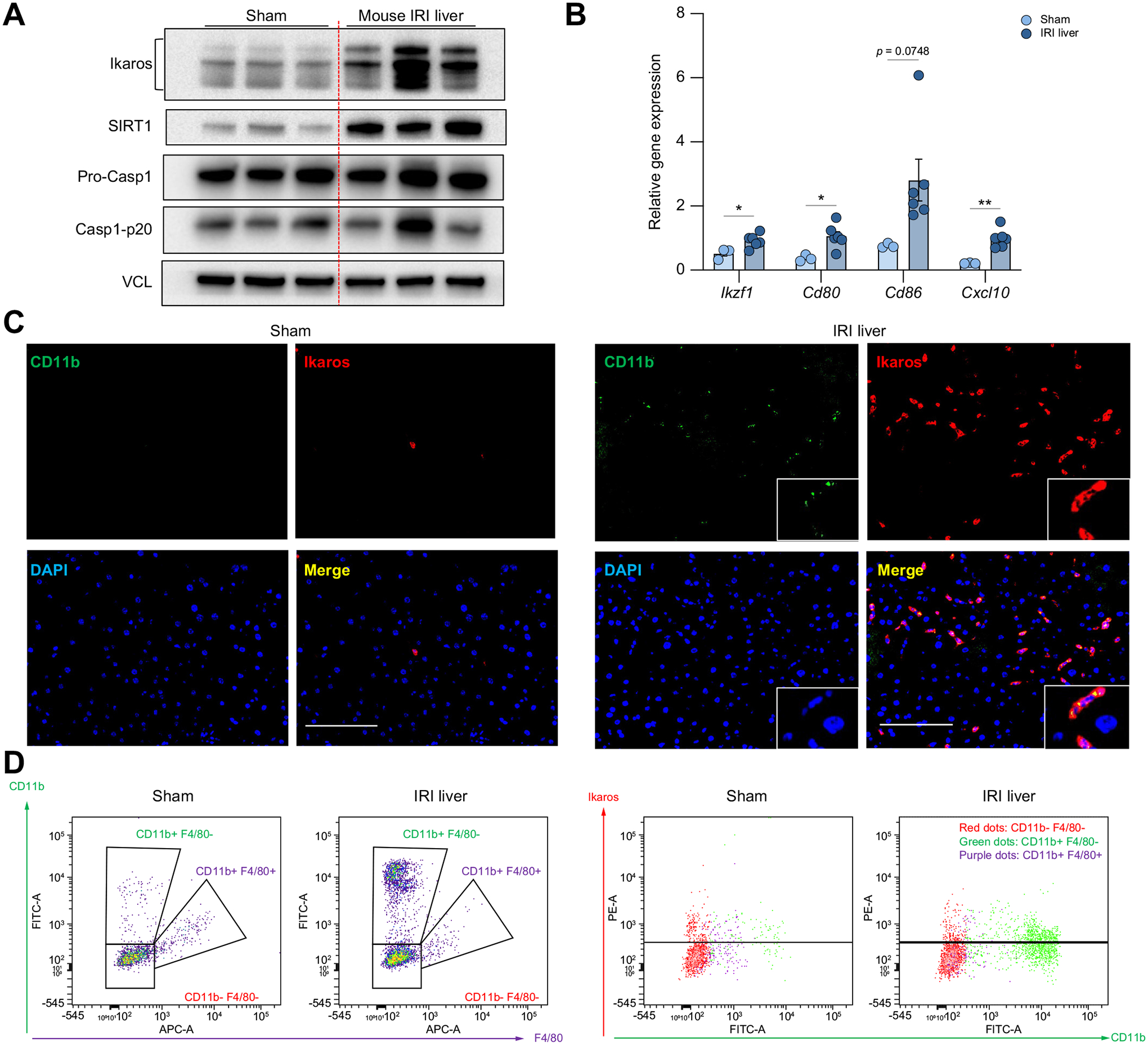
Ikaros expression by recruited macrophages in IR-stressed mouse WT liver. (A-C) Hepatocellular damage in wild-type mice subjected to 60 min portal triad blockade and 6 h reperfusion. (A) Western blot-assisted detection of Ikaros, SIRT1, Pro-Casp1 and Casp1-p20 in Sham, and IR-stressed livers, with vinculin as a loading control (n = 3/group). (B) qRT-PCR-assisted detection of *Ikzf1, Cd80, Cd86*, and *Cxcl10* gene expression in Sham (n = 3) and IR-stressed liver (n = 6). Data shown are mean±SEM. *p <0.05; **p <0.01; Student’s *t* test. (C) Representative immunofluorescence staining of CD11b (green), Ikaros (red), and merged images of Sham liver (left panel) and IR-stressed liver (right panel) (original magnification, ×400; scale bar, 100 μm; repeated 3 times with similar results). (D) Liver non-parenchymal cells were isolated from Sham and IR-stressed livers. CD11b-F4/80− cells (red dots), CD11b+F4/80− cells (green dots) and CD11b+F4/80+ cells (purple dots) were gated based on FACS staining of CD11b and F4/80 (left panel). Representative Ikaros expression of each gated population (right panel). Experiment was repeated at least 3 times with similar results. IR, ischemia-reperfusion; qRT-PCR, quantitative reverse-transcription PCR.

We next screened for Ikaros, SIRT1, and Casp1-p20 expression in a mouse model of partial liver warm ischemia (60 min). By 6 h post-reperfusion, the peak of hepatocellular damage in this model,^27^ we observed increased levels of Ikaros in IR-stressed livers associated with enhanced SIRT1 and Casp1-p20, compared with controls (Fig. 2A). The hepatic induction of *IKZF1* was accompanied by increased levels of *Cd80, Cd86*, and *Cxcl1*0 mRNA (Fig. 2B). Then, we attempted to identify the dominant Ikaros-producing cell in our model. Indeed, hepatic Ikaros co-localized with the CD11b surface marker by immunofluorescence (IF) staining (Fig. 2C), while FACS analysis revealed CD11b+F4/80− cells as the principal Ikaros-expressing non-parenchymal cells (Fig. 2D). The expression of Ikaros by CD11b+F4/80+ liver-resident cells was comparable between Sham and IR-stressed groups (Fig. S1D). Furthermore, extending the hypoxia time (from 60 min to 90 min) increased the hepatic recruitment of Ikaros-expressing CD11b+ cells (Fig. S2). These results identify infiltrating macrophages as the major contributor to Ikaros expression in IR-stressed mouse liver.

### Ikaros silencing suppresses inflammasome activation and upregulates SIRT1 in BMMs

**Fig. 3.**
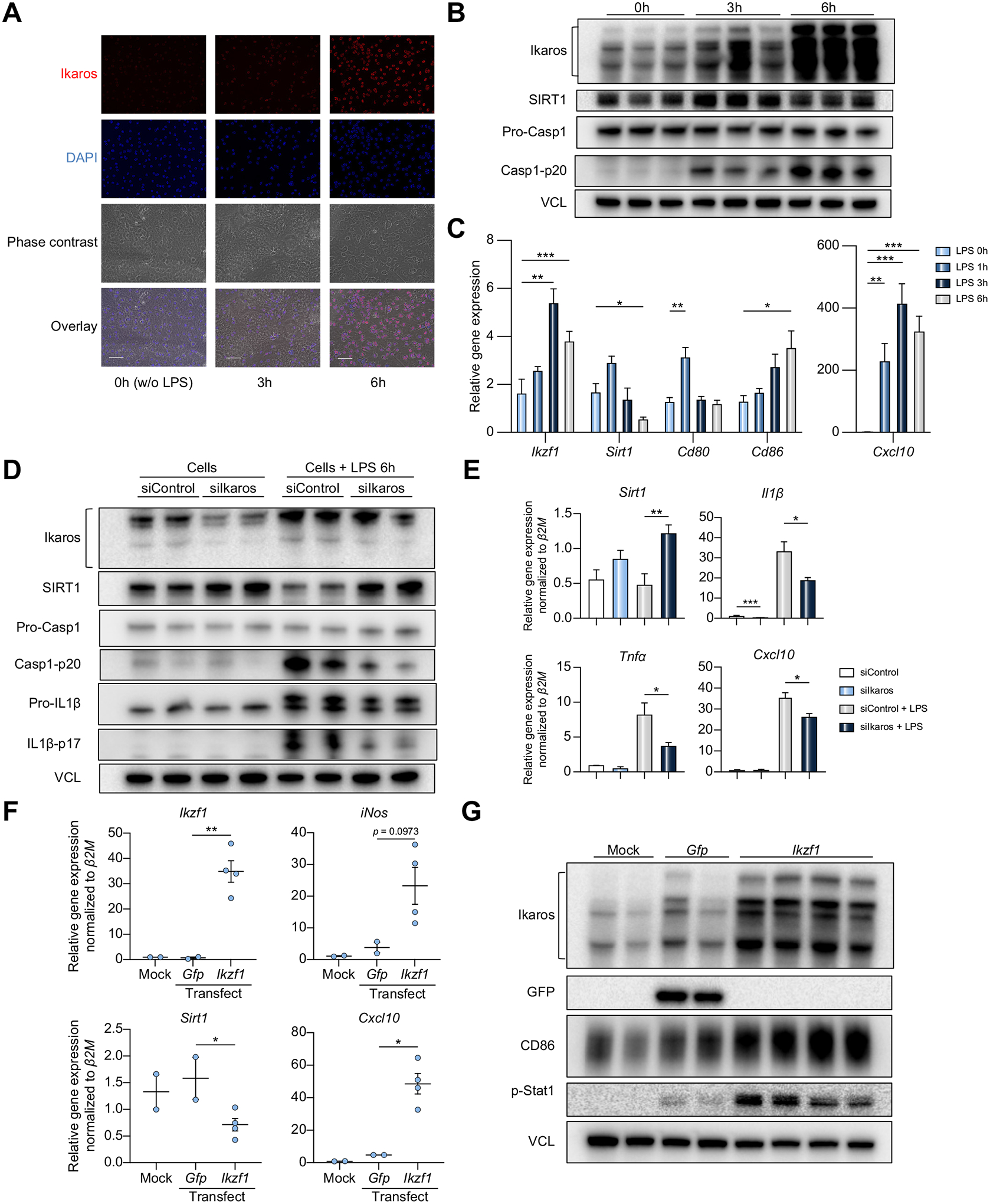
Ikaros silencing suppresses macrophage inflammation/caspase-1 signaling and upregulates SIRT1. (A-C) Wild-type mouse BMM cultures were stimulated with LPS (100 ng/ml, 0–6h). (A) Immunofluorescence staining of Ikaros (red) (original magnification, ×400; scale bar, 50 μm). (B) Total lysates from LPS-conditioned BMM (0–6 h) were probed by western blots for Ikaros, SIRT1, Pro-Casp1, Casp1-p20, and VCL as a loading control (n = 3). (C) qRT-PCR-assisted detection of mRNA coding for *Ikzf11, Cd80, Cd86, Cxcl10* in LPS-stimulated BMMs (n = 4/group). Expression levels were normalized to *Gapdh*. (D-E) Mouse (C57BL/6) BMMs transfected with siControl or siIkaros RNAs were stimulated with LPS (100 ng/ml, 6 h). (D) Lysates from BMMs were probed for Ikaros, SIRT1, Pro-Casp1, Casp1-p20, Pro-IL1β, IL1β-p17, and VCL as a loading control. (E) qRT-PCR-assisted analysis of *Sirt1, Il1β, Tnfα*, and *Cxcl10* normalized by β2M (n = 3/group). (F-G) Mouse (C57BL/6) BMMs were transfected with *Ikzf1* mRNA or *GFP* mRNA. (F) qRT-PCR-assisted analysis of *Ikzf1, Sirt1, Cxcl10, iNos* expression. Data were normalized to β2M (n = 2/Mock and *GFP*-transfected groups; n = 4/*Ikzf1*-transfected group). (G) Lysates from transfected BMMs were probed by western blots for expression of Ikaros, GFP, CD86, p-STAT1, with VCL as a loading control. Data shown are mean ± SEM. *p <0.05; **p <0.01; ***p <0.001, Student’s *t* test. BMM, bone marrow-derived macrophage; LPS, lipopolysaccharide; qRT-PCR, quantitative reverse-transcription PCR; si(RNA), small-interfering RNA.

To focus on macrophage-specific Ikaros function, we next investigated primary mouse bone marrow-derived macrophage (BMM) cultures. LPS stimulation (100 ng/ml for 6 h) increased mRNA levels of *Izkf1* and inflammation markers (Fig. 3C), and subsequently translation of the Ikaros protein. This was accompanied by upregulation of Casp1-p20, as assessed by IF and western blots, respectively (Fig. 3A,B and S3A). Then, we employed the small-interfering RNA (siRNA) approach to test whether Ikaros signaling regulates macrophage inflammation *in vitro*. BMMs transfected with siRNAs to suppress the Ikaros gene (siIkaros) or scrambled siRNAs (siControl), were stimulated with LPS. We found that Ikaros silencing increased SIRT1 expression, accompanied by decreased Casp1-p20 and IL1β-p17 levels (Fig. 3D and S3B). At the same time, we observed an increase in *Sirt1* mRNA (*p* <0.01), which correlated with decreased pro-inflammatory cytokine programs (*Tnfα, Il1β, Cxcl10*; *p* <0.05) compared with siControl-BMM (Fig. 3E). To determine whether overexpression of Ikaros influences macrophage inflammation, we cultured mouse BMMs transfected with *Ikzf1* mRNA or *GFP* mRNA as a control. Unlike Ikaros-silenced BMMs, those transfected with *Ikzf1* mRNA displayed increased Ikaros, CXCL10, and pro-inflammatory markers (iNos, p-STAT1, CD86) but decreased *Sirt1* mRNA compared to controls (Fig. 3F/G/S3C). These results highlight that the regulatory function of Ikaros in macrophage inflammation is negatively associated with SIRT1 signaling.

### Ikaros regulates BMM pyroptosis in a SIRT1-dependent manner

**Fig. 4.**
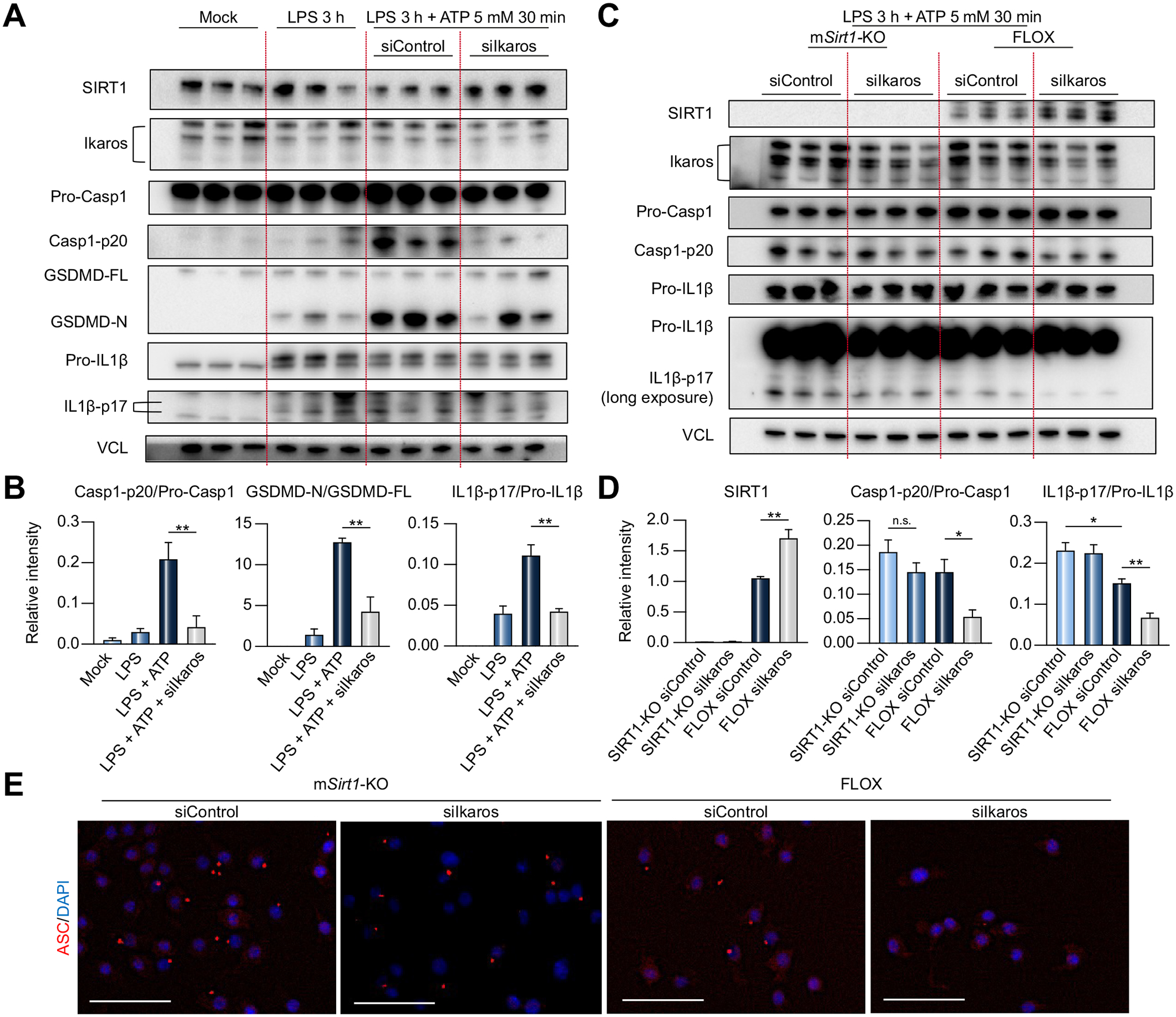
Ikaros regulation of inflammasome activation in BMMs is SIRT1 dependent. (A-B) BMMs from wild-type mice transfected with siIkaros or siControl RNAs were stimulated with LPS (1 μg/ml, 3 h), followed by ATP (5 mM, 30 min). (A) Lysates were probed by western blots for expression of SIRT1, Ikaros, Pro-Casp1, Casp1-p20, GSDMD-FL, GSDMD-N, Pro-IL1β, IL1β-p17 and VCL as a loading control. (B) Relative intensity ratios with VCL normalization. (C-D) BMMs from FLOX and m*Sirt1*-KO mice transfected with siIkaros or siControl RNAs were stimulated with LPS, followed by ATP. (C) Lysates were probed by western blots for expression of SIRT1, Ikaros, Pro-Casp1, Casp1-p20, Pro-IL1β, IL1β-p17 and VCL as a loading control. (D) Relative intensity ratios with VCL normalization. Data shown are mean ± SEM; n = 3/group. *p <0.05; **p <0.01 by Student`s *t* test. (E) Representative immunohistochemical detection of ASC (red) and DAPI (blue) in FLOX and m*Sirt1*-KO stimulated with LPS/ATP (original magnification, ×400; scale bar, 50 μm). BMM, bone marrow-derived macrophage; KO, knockout; LPS, lipopolysaccharide; si(RNA), small-interfering RNA.

To determine whether Ikaros signaling might regulate pyroptosis, an inflammatory programmed cell death platform, we transfected WT BMMs with siIkaros or siControl RNAs, followed by LPS/ATP stimulation. Indeed, siIkaros RNA-conditioned WT BMMs exhibited increased SIRT1 but decreased Casp1-p20 (*p* <0.01), GSDMD-N (*p* <0.01), and IL1β (*p* <0.01) levels compared with siControl RNA group (Fig. 4A,B). Notably, Ikaros silencing triggered a remarkable decrease of secreted Casp1-p20, IL1β, and IL18 in BMM supernatants (Fig. S4A). Having confirmed the relationship between the Ikaros-SIRT1 axis and pyroptosis under LPS stimulation (Fig. S5), we then conditioned BMMs from FLOX *vs*. m*Sirt1*-KO mice with siIkaros or siControl RNAs, followed by LPS/ATP stimulation. Indeed, Ikaros silencing increased SIRT1 expression in FLOX BMMs (*p* <0.01). While m*Sirt1*-deficient BMMs showed increased IL1β maturation (*p* <0.05), Ikaros silencing in m*Sirt1*-deficient but not *Sirt1*-proficient (FLOX) BMMs failed to reduce IL1β (Fig. 4C,D). Additionally, Ikaros silencing suppressed the formation of ASC (apoptosis-associated speck-like protein containing a CARD) speck in a SIRT1-dependent manner (Fig. 4E). Of note, there were no clear differences in the expression of NACHT, LRR, and PYD domain-containing protein 3 (NLRP3) between the groups, while Ikaros silencing increased iNos in FLOX but not m*Sirt1*-KO BMM cultures (Fig. S4). These data show that Ikaros regulates the canonical inflammasome-pyroptosis pathway in a SIRT1-dependent manner.

### Myeloid Ikaros knockdown attenuates liver IRI by suppressing the inflammasome-pyroptosis pathway while promoting SIRT1 activation in CD11b-DTR mouse

**Fig. 5.**
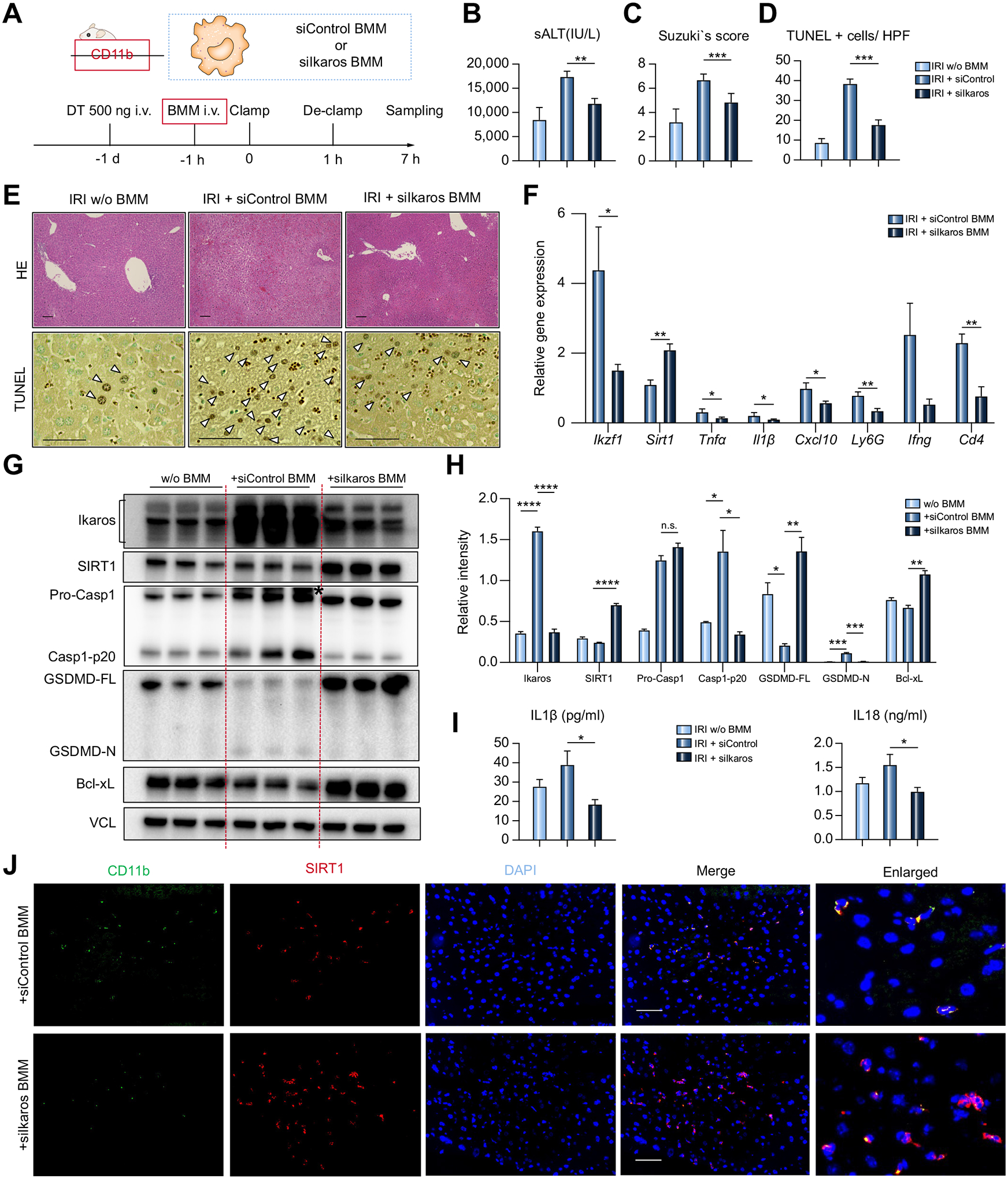
Adoptively transferred Ikaros-silenced BMMs attenuate liver IRI in CD11b-DTR mice by upregulating SIRT1 and suppressing caspase-1-GSDMD processing. CD11b-DTR mice untreated or reconstituted with BMMs transfected with siControl *vs*. siIkaros RNAs were subjected to 60 min warm ischemia and 6 h reperfusion. (A) Workflow of CD11b+ cell depletion/reconstitution by BMMs, followed by warm liver IRI. (B) sALT levels (n = 5–6/group). (C) Suzukìs histological grading of liver IRI and (D) quantification of TUNEL+ cells/HPF (n = 5–6/group). (E) Representative H&E (original magnification, ×100; scale bar, 100 μm) and TUNEL (original magnification, ×400; scale bar, 50 μm) staining. Arrowheads show TUNEL+ cells. (F) qRT-PCR-assisted detection of mRNA coding for *Ikzf1, Sirt1, Tnfα, Il1β, Cxcl10, Ly6G, Ifnγ*, and *Cd4*. Data were normalized to *Gapdh* expression (n = 3–4/group). (G) Western blot-assisted detection of Ikaros, SIRT1, Pro-Casp1, Casp1-p20, GSDMD, Bcl-xL, and VCL. Asterisk indicates non-specific band. (H) Relative intensity ratios with VCL normalization (n = 3/group). (I) Serum IL1β/IL18 levels (n = 5–6/group). (J) Representative immunohistochemical images illustrate hepatic detection of CD11b (green), SIRT1 (red) in CD11b-DTR mice repopulated with siControl or siIkaros BMM (original magnification, ×400, scale bar, 50 μm). Data shown are mean ± SEM. *p <0.05; **p <0.01; ***p <0.001; ****p <0.0001 by Student’s *t* test. BMM, bone marrow-derived macrophages; HPF, high power field; IRI, ischemia-reperfusion injury; LPS, lipopolysaccharide; qRT-PCR, quantitative reverse-transcription PCR; sALT, serum alanine aminotransferase; si(RNA), small-interfering RNA.

To focus on the impact of myeloid-specific Ikaros in the mechanism of liver IRI, we next employed the CD11b-DTR adoptive transfer mouse model.^26^ After pretreatment with DT (200 ug/kg i.v. day −1) to deplete CD11b+ native macrophages, mice were challenged with *in vitro* generated siRNA-conditioned BMMs (5×10^5^ cells i.v.) at 1 h before the hepatic IR-insult (Fig. 5A). The depletion and repopulation of CD11b+ cells were confirmed by flow cytometry, while adjunctive BMM transfer markedly increased Ikaros and Casp1-p20 levels in IR-stressed livers (Fig. S6). Infusion of BMMs conditioned with siIkaros ameliorated liver IRI. This was demonstrated by diminished sALT (siControl = 17,342±1,205 *vs*. siIkaros = 11,797±1,101 IU/L, *p* = 0.0094; Fig. 5B); decreased Suzukìs score (siControl = 6.667±0.2108 *vs*. siIkaros = 4.833±0.3073, *p* = 0.0006; Fig. 5C); depressed TUNEL+ cells/high power field (HPF) (siControl = 38.33±1.45 *vs*. siIkaros= 17.67±1.453, *p* = 0.0005; Fig. 5D,E); and histological preservation of the hepatic architecture (Fig. 5E). As shown in Fig. 5F,G, adoptive transfer of siControl-BMMs elevated, while the infusion of siIkaros-BMMs diminished, hepatic Ikaros levels, confirming the pathogenic function of Ikaros in liver-infiltrating macrophages. qRT-PCR analyses revealed Ikaros silencing decreased hepatic pro-inflammatory phenotypes (*Tnfα, Il1β, Cxcl10, Ly6G, Ifnγ, Cd4*), while increasing *Sirt1* expression (Fig. 5F). Western blots revealed that adoptive transfer of siIkaros-conditioned BMMs decreased Casp1-p20 and GSDMD-N while increasing SIRT1 (Fig. 5G,H). Consistently, serum IL1β (siControl = 38.96±7.210 *vs*.18.40±2.557 pg/ml, *p* = 0.0276) and IL18 (siControl = 1.554±0.2276 *vs*. 0.9969±0.0885 ng/ml, *p* = 0.0370) levels were significantly reduced after silencing of Ikaros in transferred BMMs (Fig. 5I).

IR-stressed livers in CD11b-DTR mice repopulated with siIkaros- but not siControl-BMMs consistently showed increased anti-apoptotic Bcl-xL expression (Fig. 5G). Immunofluorescent staining of adoptively transferred CD11b+ BMMs in IR-stressed livers revealed higher levels of SIRT1 after Ikaros knockdown (siIkaros), suggesting Ikaros-silenced SIRT1-enriched macrophages preferentially accumulated in IR-stressed livers of CD11b-DTR test mice (Fig. 5J). We confirmed the pattern of enhanced Ikaros/increased pyroptosis (GSDMD) being negatively associated with SIRT1 in liver-infiltrating CD11b+ cells in a murine LPS/D-Galactosamine hepatitis model (Fig. S7). These results show that myeloid Ikaros signaling mediates the canonical inflammasome-pyroptosis pathway to aggravate the acute hepatic pro-inflammatory response by negatively regulating SIRT1.

### Myeloid SIRT1 deficiency exacerbates liver IRI and promotes inflammasome-pyroptosis activation *in vivo*

**Fig. 6.**
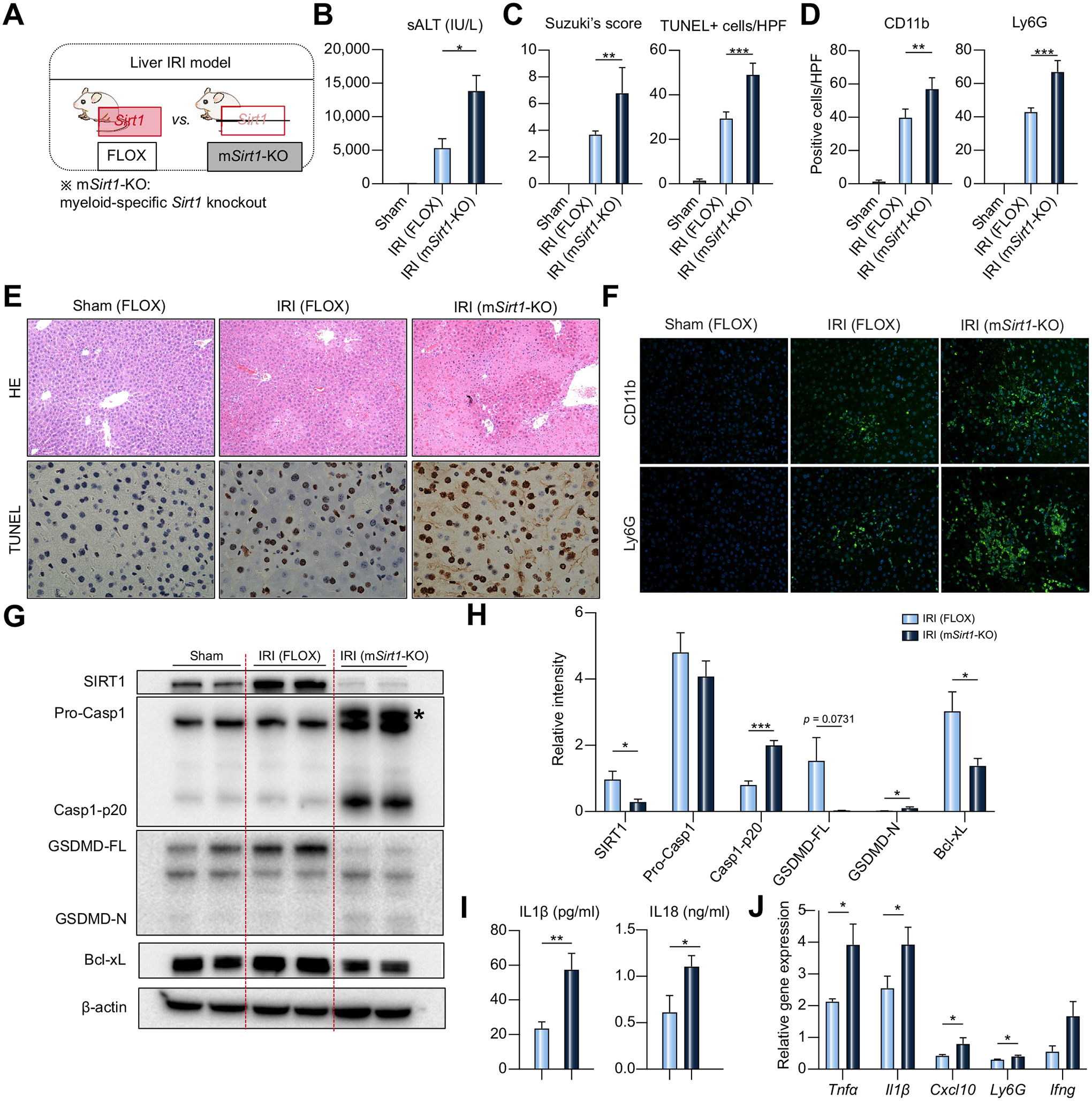
Myeloid-specific disruption of SIRT1 signaling activates caspase-1-GSDMD processing and exacerbates innate inflammation in mouse liver IRI. (A) FLOX control and myeloid-specific SIRT1-deficient (m*Sirt1*-KO) mice were subjected to warm liver IRI, followed by liver/serum sampling at 6 h. (B) sALT levels (n = 5–6/group). (C) Suzukìs histological grading of liver IRI and quantification of TUNEL+ cells/HPF (n = 5–6/group). (D) Quantification of IR-infiltrating CD11b+, Ly6G+ cells (positive cells/HPF; n = 5/group). (E) Representative H&E staining (original magnification, ×100) and TUNEL staining (original magnification, ×400). (F) Representative immunohistochemistry of liver-infiltrating CD11b+, Ly6G+ cells (original magnification, ×400). (G) Representative western blot-assisted detection of SIRT1, Pro-Casp1, Casp1-p20, GSDMD, Bcl-xl, and β-actin. Asterisk indicates non-specific band. (H) Western blot-assisted quantification of SIRT1, Pro-caspase1, Casp-p20, GSDMD-FL, GSDMD-N, Bcl-xl with β-actin normalization (n = 5–6/group). (I) Serum IL1b and IL18 levels (n = 5–6/group). (J) qRT-PCR-assisted detection of mRNA coding for *Tnfα, Il1β, Cxcl10, Ly6G*, and *Ifnγ*. Data were normalized to *Gapdh* expression (n = 4–5/group). Data shown are mean ± SEM. *p <0.05; **p <0.01, ***p <0.001 by Student’s *t* test. HPF, high power field; IRI, ischemia-reperfusion injury; qRT-PCR, quantitative reverse-transcription PCR; sALT, serum alanine aminotransferase; si(RNA), small-interfering RNA.

Having documented the significance of the Ikaros–SIRT1 axis in macrophage activation *in vitro*, we then asked whether myeloid-specific SIRT1 regulates macrophage function in a murine liver IRI model. Groups of m*Sirt1*-deficient (KO) and *Sirt1*-proficient (FLOX) mice were subjected to 60 min hepatic warm ischemia (Fig. 6A). By 6 h of reperfusion, m*Sirt1*-KO mice displayed higher sALT levels (control = 5,324±1,398 *vs*. m*Sirt1*-KO = 13,851±2,295 IU/L, *p* = 0.0210; Fig. 6B); augmented Suzuki’s histological score of hepatocellular damage (control = 3.683±0.2725 *vs*. m*Sirt1*-KO = 6.780±0.8570, *p* = 0.0047; Fig. 6C); and increased frequency of hepatic TUNEL+ cells (control = 29.38±1.22/HPF *vs*. m*Sirt1*-KO = 49.00±2.34/HPF, *p* <0.0001; Fig. 6C,E). The disruption of myeloid-specific SIRT1 enhanced liver infiltration by macrophages and neutrophils (Fig. 6D,F), evidenced by IF staining/quantification of sequestered CD11b macrophages (control = 39.78±2.13/HPF *vs*. m*Sirt1*-KO = 56.98±3.07/HPF, *p* = 0.0011) and Ly6G neutrophils (control = 42.87±2.486/HPF *vs*. m*Sirt1*-KO = 66.98±3.00/HPF, *p* = 0.0001). Western blot-assisted analysis and ELISA data revealed mSIRT1-deficiency increased hepatic Casp1-p20 and GSDMD-N as well as serum IL1β (control = 23.51±3.890 *vs*. m*Sirt1*-KO=57.46±9.396 pg/ml, *p* = 0.0047) and IL18 (control = 0.6143±0.1784 *vs*. m*Sirt1*-KO=1.1106±0.1176 ng/ml, *p* = 0.0375) levels (Fig. 6G–I). Moreover, qRT-PCR analysis showed an increased pro-inflammatory gene expression program (*Tnfα, Il1β, Cxcl10, Ly6G*, and *Ifnγ*) (Fig. 6J), while Bcl-xL protein levels associated with hepatoprotection were reduced in m*Sirt1*-KO recipients (Fig. 6G,H). Furthermore, a profound suppression of phosphorylated NLRP3 at Ser295 (p-NLRP3) was detected selectively in m*Sirt1*-KO, despite comparable total NLRP3 levels in both FLOX and m*Sirt1*-KO livers (Fig. S8A–C). These results are consistent with the homeostatic function of myeloid SIRT1, evidenced by suppressed pyroptosis, diminished pro-inflammatory cell activation/infiltration, and mitigated hepatocellular injury in IR-stressed livers.

### Negative regulation of SIRT1 by Ikaros is AMPK-dependent

**Fig. 7.**
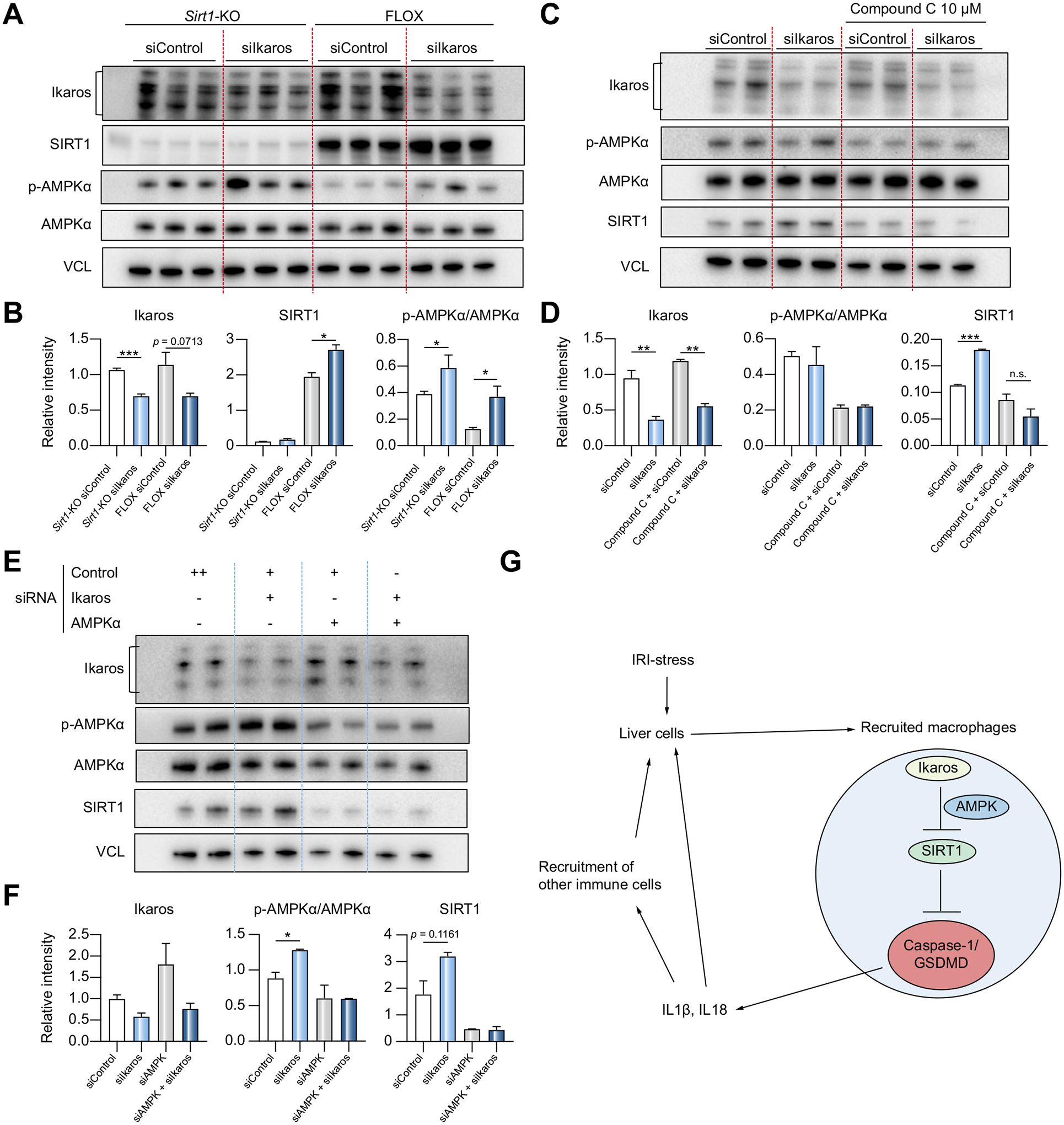
Negative regulation of SIRT1 by Ikaros is AMPK dependent. (A) BMMs from m*Sirt1*-KO and FLOX mice were transfected with siControl or siIkaros RNAs. Lysates were probed by western blots for Ikaros, SIRT1, p-AMPKα, AMPKα and VCL as a loading control. (B) Relative intensity ratios with VCL normalization (n = 3). (C) wild-type BMMs transfected with siControl or siIkaros RNAs were pre-treated with Compound C (10 μM, 2 h). Lysates were probed by western blots for Ikaros, p-AMPKα, AMPKα, SIRT1 and VCL expression. (D) Relative intensity ratios with VCL normalization (n = 2). (E) Wild-type BMMs were transfected with siAMPKα/siControl RNAs for 48 h, followed by another 24 h transfection with siIkaros/siControl RNAs. Lysates were probed by western blots. (F) Relative intensity ratios with VCL normalization (n = 2). (G) Schematic illustration how myeloid-specific Ikaros–SIRT1 axis orchestrates macrophage inflammation. Ikaros negatively regulates SIRT1 via AMPK, which inhibits caspase-1/GSDMD processing. In the acute liver IRI-phase, Ikaros signaling favors sterile inflammation in conjunction with negative regulation of SIRT1 via AMPK, resulting in activation of the canonical inflammasome-pyroptosis pathway to release IL1β and IL18. (A-F) Data shown are mean ± SEM. *p <0.05; **p <0.01, ***p <0.001 by Student’s *t* test. BMM, bone marrow-derived macrophages; IRI, ischemia-reperfusion injury; KO, knockout; si(RNA), small-interfering RNA.

Consistent with B-lymphoid studies,^22^ we found upregulated phosphorylated AMPKα (p-AMPKα) after Ikaros knockdown in both FLOX and m*Sirt1*-KO BMM cultures (Fig. 7A,B). This indicates Ikaros regulates AMPKα expression before SIRT1 activation. To investigate whether Ikaros controls SIRT1 through AMPK signaling, we pre-treated BMMs with a selective AMPK inhibitor (Compound C), followed by siIkaros RNA supplement. Indeed, pharmacological AMPK inhibition prevented SIRT1 increase after Ikaros silencing (Fig. 7C,D). Furthermore, adjunctive siAMPKα RNA transfection failed to affect otherwise readily upregulated SIRT1 seen after Ikaros silencing alone (Fig. 7E,F). Hence, Ikaros regulates SIRT1 through AMPK activation.

## Discussion

In this study, we identified Ikaros–SIRT1 signaling as a regulator of the canonical macrophage inflammasome-pyroptosis pathway, leading to distinct signatures of sterile inflammation in mouse and human IR-stressed livers. To dissect the impact of the Ikaros–SIRT1 axis on macrophage activation, we analyzed mouse BMM cultures (under Ikaros silencing), coupled with screening of IR-stressed livers in WT, myeloid-specific *Sirt1*-KO, and macrophage-repopulated CD11b-DTR mouse recipients. Parallel screening of hepatic biopsies from 55 liver transplant recipients underlines the role of Ikaros–SIRT1 regulation in the pro-inflammatory myeloid profile and inflammasome-pyroptosis pathway in human liver stress/injury.

As Ikaros was expressed primarily by liver-infiltrating CD11b+ macrophages (Fig. 2C,D), we screened for Ikaros function in BMM cultures (Fig. 4). Ikaros silencing impaired BMM commitment toward the pro-inflammatory phenotype and suppressed the inflammasome-pyroptosis pathway, with simultaneous upregulation of SIRT1. Notably, SIRT1 ablation abrogated the Ikaros-stimulating effect on caspase-1-GSDMD processing, a prerequisite for pyroptosis. These *in vitro* data highlight the novel function of Ikaros-SIRT1 in regulation of the inflammasome.

To focus on the Ikaros–SIRT1 axis in BMM activation/recruitment *in vivo*, we utilized the CD11b-DTR mouse system, in which: i) adoptively transferred Ikaros-proficient BMMs recreated liver damage in otherwise IRI-resistant CD11b-deficient recipients; ii) Ikaros-silenced BMMs ameliorated liver IRI/pyroptosis, compared with Ikaros-proficient controls; and iii) SIRT1 was readily expressed by adoptively transferred liver-infiltrating Ikaros-silenced CD11b+ BMMs. In addition to comparable SIRT1-pyroptosis signatures *in vitro* and *in vivo*, these data document the divergent impact of Ikaros signaling upon hepatic inflammation. Together, we propose that Ikaros signaling drives activation of the inflammasome and exacerbates liver IRI in conjunction with negative SIRT1 regulation. Indeed, disruption of myeloid-specific SIRT1 unmasked increased pyroptosis and aggravated liver IRI (Fig. 6). However, as m*Sirt1*-KO mice underwent Cre-mediated *Sirt1* deletion under the control of M lysozyme, we cannot exclude a possibility that SIRT1 deficiency in liver-resident Kupffer cells may have also contributed to hepatic IRI or pyroptosis in liver-recruited macrophages. Of note, IR-stressed livers without BMM reconstitution showed upregulated Casp1-p20 levels compared to Sham controls (Fig. S6). Likewise, although it remains controversial whether bioactive IL1β derives from circulating monocytes or cirrhotic liver tissue itself, recent data point towards the importance of its serum levels as a prognostic marker.^28,29^ The importance of macrophage recruitment for the SIRT1-pyroptosis axis was supported by enhanced SIRT1 levels and reduced hepatic pyroptosis in siIkaros BMM-transferred DTR test mice. We have shown Kupffer cell frequency drastically decreases after IR stress, with a simultaneous hepatic influx of circulating CD11b+ macrophages.^30^ Notably, we have detected upregulated Ikaros levels in liver-infiltrating CD11b+ cells, coinciding with enhanced pyroptosis (GSDMD) but negatively associated with SIRT1, in a murine LPS/D-Galactosamine hepatitis model (Fig. S7). These findings validate the role of the Ikaros–SIRT1 axis in the mechanism of macrophage pyroptosis and innate immune-driven liver inflammation cascade.

We have reported on the activation of multiprotein inflammasome complex in the context of caspase-1 induced IL1β production in the mechanism of liver IRI.^7,8^ Although caspase-1 may induce apoptosis rather than pyroptosis in certain cell types,^31^ accumulating evidence points to GSDMD, the substrate of caspase-1, as a critical mediator of pyroptosis.^6^ Indeed, in the current study, upregulated caspase-1 in SIRT1-deficient BMMs was mediated by GSDMD cleavage both *in vitro* and *in vivo*. Recently, caspase1-GSDMD processing was demonstrated in innate immune cells but not in hepatocytes, indicating that selective blockade of inflammasome-activated pyroptosis in the liver infiltrate might mitigate IRI.^9^ Our current study also showed that the myeloid Ikaros-SIRT1 axis regulated caspase-1-GSDMD processing to secrete IL1β and IL18, *i.e*. cytokines central to inflammasome activation and pyroptosis.

In agreement with the ability of bound IL18 to reduce liver infiltration by neutrophils and CD3+ cells,^32^ *IFNγ* and *CD4* gene expression showed a similar trend in our study, albeit these failed to reach statistical significance. Thus, we may link the pro-inflammatory profile in cultured BMM/IR-stressed liver with upregulated caspase-1-GSDMD processing and IL1β/IL18 secretion. However, caspase-1 may exert a pro-inflammatory function independent of IL1β/IL18,^33^ while GSDMD-dependent release of these cytokines might occur without macrophage death.^34,35^ Hence, further studies are needed to clarify the relationship between pro-inflammatory signaling and caspase-1-GSDMD processing in the pyroptotic pathway in liver IRI.

In addition to IL1β, IL1α was recently found to activate the inflammasome platform during cirrhosis.^29^ In agreement with the distinct cleavage mechanism and IL1β/IL18 release,^36,37^ our LPS/ATP-stimulated BMM cultures showed the release of pro-IL1α, but not of its mature (C terminal) form. Future experiments need to address the exact role of IL1α processing and signaling in sterile inflammation in response to IR stress.

SIRT1 signaling extends lifespan and protects cells from various environmental stresses.^38^ We reported that treatment with resveratrol, a small molecule SIRT1 activator, promoted myeloid protective function in mouse liver IRI.^18,19^ In the present study, we contrasted myeloid-specific SIRT1 signaling to demonstrate, for the first time, that SIRT1 activation suppressed pyroptosis in IR-stressed livers. This is consistent with its effects on macrophage inflammasome function^39^ when the myeloid AMPK/SIRT1 axis suppressed colitis through NLRP3 inflammasome and caspase-1 activation. Although SIRT1 induction has been linked with macrophage inflammasome activation, the regulatory mechanism of SIRT1 transcription has not been studied. Here, we report a new molecular mechanism by which Ikaros negatively regulates SIRT1 in a mouse model of hepatic inflammation and human OLT.

Crosstalk between immunity and metabolism is increasingly recognized,^40^ and Ikaros signaling was shown to affect the AMPK metabolic pathway in a leukemia study.^23^ Notably, both pharmacological and genetic inhibition of AMPK in our study point towards Ikaros-SIRT1 function being AMPK dependent. Although SIRT1 and AMPK are metabolic stress sensors, which may regulate each other in a positive feedback loop,^41,42^ putative regulation of AMPK by SIRT1 remains unclear.^43^ Indeed, consistent with published data from *Sirt1* KO mice,^44^ m*Sirt1*-deficient BMMs showed higher expression of p-AMPKα than FLOX counterparts in our study. In this context, p-AMPKα upregulation alone does not necessarily suppress inflammasome activation under stress. Taken together, as part of an orchestrated molecular communication network, Ikaros regulates macrophage activity via the AMPK-SIRT1 axis. With published data deriving primarily from lymphocyte differentiation/developmental studies, there is an unmet need to further dissect Ikaros–SIRT1 signaling pathways in activated macrophages.

The question arises as to how the Ikaros-SIRT1 axis may regulate the canonical inflammasome-pyroptosis pathway. First, Ikaros negatively associated with phosphorylation of NLRP3 at Ser295 in our study, which was shown by others to inhibit inflammasome assembly.^45^ Notably, Ikaros knockdown did not affect total NLRP3 levels despite suppressing NLRP3 activation in our LPS/ATP macrophage culture system. Thus, consistent with recent findings,^46^ the Ikaros-SIRT1 axis may control inflammasome activation through post-translational modifications. In contrast to SIRT1, sirtuin 2 regulates the acetylation of NLRP3.^47^ Thus, phosphorylation of NLRP3 may represent the molecular target for the Ikaros-SIRT1 axis. Second, Ikaros negatively regulated iNos (Fig. S4), which was shown to suppress inflammasome activation.^48,49^ Furthermore, in line with unchanged total NLRP3 levels after Ikaros knockdown in our study, others suggested post-translational modulation as a mechanism by which iNos regulates inflammasome signaling.^48,50^ On the other hand, the Ikaros-SIRT1 axis failed to correlate with anti-inflammatory IL10 signaling (data not shown), which was reported by others to regulate NLRP3 function.^51^

In our clinical arm, it was remarkable to find that increased Ikaros levels were accompanied by significantly increased inflammatory markers and cleaved caspase-1 but decreased SIRT1 expression in human OLT biopsies (Fig. 1). Thus, Ikaros might represent a novel regulator of the macrophage inflammasome-pyroptosis pathway in humans, while both Ikaros and SIRT1 might serve as putative therapeutic targets in liver transplantation. One might envision that prospective transplant patients may be conditioned, if needed, with Ikaros-suppressing or SIRT1-inducing compounds before the surgical procedure. Consistent with our reports on genetically modified BMM transfers in IRI-OLT models,^26,52^ native BMMs can be manipulated *ex vivo* to suppress Ikaros or enhance SIRT1 expression and then be autografted back into patients.

Human OLT recipients with Ikaros-high levels had significantly higher BMI than the Ikaros-low clinical cohort. Additionally, the ratios of metabolic diseases such as diabetes mellitus and hypertension were more common in the Ikaros-high patient group, although differences failed to reach statistical significance (Table S1). As the number of obese patients undergoing OLT increases,^53^ several studies focused on pre-transplant obesity as a risk factor, while recipient BMI as a prognostic factor for worsened OLT outcomes remains controversial.^54^ The relationship between obesity and macrophage activation was studied in non-alcoholic fatty liver disease and non-alcoholic steatohepatitis.^55^ In these conditions, distant macrophage differentiation in adipose tissues is regulated through cytokines, damage-associated molecular patterns, or free fatty acids.^56^ Furthermore, peripheral blood-derived macrophages from individuals with type 2 diabetes displayed enhanced caspase-1 activation.^57^ Therefore, patients with increased Ikaros expression might present activated inflammasome and pyroptotic circulating monocyte/macrophage profiles. Despite apparent disparities in pro-inflammatory parameters and IR-mediated hepatocellular damage, other factors, such as patient/graft survival or early allograft dysfunction, did not show significant differences. First, as the number of liver biopsies was limited (n = 55), a prospective study in a larger patient cohort is warranted. Second, the predictive value of Ikaros for post-OLT clinical outcomes remains uncertain and needs to be examined.

In summary, our translational study documents a novel regulatory function of the Ikaros–SIRT1 axis via AMPK signaling in macrophage activation. It may serve as a therapeutic target and as a checkpoint regulator of homeostasis in response to acute hepatic stress/injury in mouse and human livers.

## Supplementary Material

1

2

3

## Data Availability

The authors declare that all data supporting the findings of this study are available in the article or supplementary information file.

## Highlights

- Ikaros transcription factor is expressed by host macrophages recruited into ischemia-stressed mouse and human livers.
- Ikaros regulates macrophage inflammasome activation and sterile liver inflammation via AMPK-SIRT1 signaling.
- Ikaros regulates the inflammasome-pyroptosis response in stressed macrophages in a SIRT1-dependent manner.
- Increased Ikaros levels accompanied by inflammasome signaling correlate with worse human liver transplant function.
- Ikaros-SIRT1 axis may serve as pyroptosis biomarker and homeostasis checkpoint against liver injury in mice and humans.

## Abbreviations

AMPK: AMP-activated protein kinase
BMM: bone marrow-derived macrophages
GSDMD: Gasdermin D, HPF, high power field
IF: immunofluorescence
IKZF1: Ikaros family zinc finger protein 1
IRI: ischemia-reperfusion injury
KO: knockout
LPS: lipopolysaccharide
OLT: orthotopic liver transplantation
qRT-PCR: quantitative reverse-transcription PCR
sALT: serum alanine aminotransferase
sAST: serum aspartate aminotransferase
siRNA: small-interfering RNA
SIRT1: sirtuin 1
TLR4: Toll-like receptor 4
VCL: vinculin
WT: wild-type
