# Supplementary material for: Myeloid Ikaros–SIRT1 signaling axis regulates hepatic inflammation and pyroptosis in ischemia-stressed mouse and human liver
Source: J Hepatol. Author manuscript; Available in PMC 2022 Nov 28. (PMC9704689; doi:10.1016/j.jhep.2021.11.026)
Supplement: 1 [file NIHMS1852203-supplement-1.pdf]

Myeloid Ikaros–SIRT1 signaling axis regulates hepatic inflammation and pyroptosis in ischemia-stressed mouse and human liver

Kentaro Kadono, Shoichi Kageyama, Kojiro Nakamura, Hirofumi Hirao, Takahiro Ito, Hidenobu Kojima, Kenneth J. Dery, Xiaoling Li, Jerzy W. Kupiec-Weglinski

Table of contents

Supplementary methods…………………………………………………………………........... 2

Fig. S1……………………………………………………………...............................……..…..6

Fig. S2………………………………………………….............................……………………......7

Fig. S3………………………………………………….............................……………………......8

Fig. S4…………………………………………………….............................…………………......9

Fig. S5…………………………………………………….............................………………......10

Fig. S6………………………………………………………….............................….........11

Fig. S7………………………………………………………………...12

Fig. S8………………………………………………………………...13

Table S1…………………………………………………………..........................……….......15

Table S2…………………………………………………………..........................……….......16

Table S3…………………………………………………………..........................……….......17

Table S4…………………………………………………………..........................……….......18

Supplementary references………………………………………………………….........19

Supplementary methods

Animals

The SIRT1 allele with floxed exon 4 was backcrossed six times into the C57BL/6 background and then bred with mice expressing Cre recombinase driven by the lysosome promoter to generate myeloid cell-specific SIRT1knockout littermates[1].

Mouse acute liver hepatitis model

Male mice (6-10 weeks of age) were anesthetized with isoflurane and treated with LPS (30μg/Kg) / D-Galactosamine (400mg/Kg) (GalN), as described[2]. Liver and blood samples were collected at 6 hours after the LPS/D-GalN injection.

Clinical liver transplant study

The model for end-stage liver disease (MELD) assessed the severity of liver disease. Cold ischemia time was defined as time from liver perfusion with UW solution to its removal from cold storage for implantation. Warm ischemia time was defined as the time from removal from cold storage to establishment of liver reperfusion.

Histopathology

Formalin-fixed liver sections (5μm) were stained with hematoxylin/eosin (H&E) and analyzed blindly by modified Suzuki's criteria on a scale from 0-4[3]. No necrosis, congestion, or centrilobular ballooning is given a score of 0, while severe congestion and >60% lobular necrosis is given a value of 4. Cell death in liver sections was detected by In-Situ Apoptosis Detection Kit (Clontech, San Diego, CA). Results were scored semi-quantitatively by blindly counting the number of positive cells in 10 HPF/section.

Cell cultures

Bone marrow macrophages (BMM) from mouse femurs/tibias were cultured (5×10^6/well) with 10% L929 medium for 7 days (94-99% CD11b+)[4] and treated with LPS (100ng/ml; catalog L5418-

2ML; Sigma) for 6h or LPS (1µg/ml) for 3h followed by ATP (5mM; catalog R0441; Thermo Scientific) for 30 min. The AMPK inhibitor, Compound C (catalog 171261-1MG), was purchased from EMD Millipore. In some experiments, BMM were transfected with Ikaros/AMPKα/scrambled siRNA (Santa Cruz Biochemistry) using Lipofectamine reagent (Invitrogen) in advance of LPS stimulation. To ectopically express Ikaros mRNA, mMESSAGE mMACHINE T7 Transcription Kit (Invitrogen) was used. Forward and reverse primers:

(5'-GCTAATACGACTCACTATAGGGACAGGCCACCATGGATGTCGATGAGGGTCAAGAC-3'; 5'-TTAGCTCAGGTGGTAACGATGCTC-3') were used to amplify T7-Ikaros using BMM + LPS as template. BMM were transfected with Ikaros mRNA using Lipofectamine mRNA transfection reagent (Invitrogen)[5].

Western blot assay

Proteins were extracted from tissue/cell samples, and their concentration was measured (BCA Assay Kit, Thermo-Fisher Scientific). An equal amount of protein was electrophoresed, blotted, incubated with primary Abs, secondary HRP-conjugated Abs, and developed. The relative value was normalized by Vinculin or β-actin. The following primary Abs were used to detect: Ikaros (14859/D6N9Y), SIRT1 (9475/D1D7), iNos (13120/D6B6S), Bcl-xL(2764/54H6), GFP (D5.1/2956), p-AMPKα (2535/40H9), AMPKα (2532), mouse ASC (67824/D2W8U), Vinculin (VCL) (18799/E1E9V) and β-actin (12620/D6A8) (all from Cell Signaling Technology), mouse cleaved caspase-1 p20 (Casper-1; Adipogen), NLRP3 (MAB7578; R&D), p-NLRP3 (PA5-105071; Invitrogen), human caspase-1 (ab207802), mouse Gasdermin D (ab209845), IL1β (ab9722) and mouse IL18 (ab71495) (Abcam).

To compare target protein expression in human OLT, densiometric quantification was conducted[4,6]. Briefly, one of the Bx samples expressing all target proteins was assigned as a control sample. Equal amount of protein lysate from each sample was applied to each well/gel, and the target

band intensity was expressed as relative band intensity to that of the positive control in the same gel. The target relative protein value was normalized according to β-actin intensity.

qRT-PCR analysis

RNA extracted with an RNAse Mini Kit (QIAGEN) was reverse transcribed into cDNA. qRT-PCR was performed using QuantStudio 3 (Applied Biosystems). Primers to amplify specific gene fragments are listed (Table S3/4). Target gene expressions were calculated by their ratios to the housekeeping β2M or GAPDH gene.

Immunofluorescence

Mouse liver samples were stained with rabbit rat-CD11b+ Ab (BD Biosciences), rat anti-Ly6G Ab (BD Biosciences), rabbit anti-Ikaros Ab and rabbit anti-SIRT1 Ab. Mouse macrophages were stained using goat anti-E-Cadherin Ab (AF748; R&D) and rabbit anti-GSDMD Ab. Signals were visualized with secondary Alexa Fluor Abs (Invitrogen, Thermo Fisher Scientific). Hepatic CD68^{+}Ly6G^{+} cells were scored semiquantitatively by counting cells in 10 HPF/section (×400).

Flow cytometry

Liver nonparenchymal cells (NPCs) were isolated, as described[7]. Briefly, liver was perfused with collagenase (MilliporeSigma Co., St. Louise, MO) solution using peristatic pump via inferior vena cava and tissue samples were pressed against a 40μm nylon cell strainer. Isolated liver NPCs were incubated with anti-mouse CD16/32 antibody (clone:93 BioLegend, San Diego, CA) to block Fc-mediated nonspecific Ab binding. Thereafter, cells were stained with the fluorochrome-conjugated antibodies; CD11b-FITC, F4/80-APC or iso-type controls (BioLegend) (Fig.S2). For Ikaros staining, cells were fixed, permeabilized, and incubated with the fluorochrome-conjugated antibodies; Ikaros-PE (BioLegend) according to the manufacturer`s instruction. Multi-parameter flow cytometric analysis was performed using a LSR Fortessa X-20 SORP (BD Bioscience, San Jose, CA) and results were

analyzed using BD FACSDiva software (BD) at the UCLA Jonsson Comprehensive Cancer Center (JCCC) and Center for AIDS Research Flow Cytometry Core Facility (UCLA, CA).

Enzyme-linked immunosorbent assay (ELISA)

Serum concentrations of IL1β and IL18 were measured with ELISA kits (Thermo-Fisher Scientific) according to the manufacturer`s protocol.

Statistical analysis

For human data, continuous values were analyzed by the Mann-Whitney *U* test and categorical variables by Fisher`s exact test. Spearman`s correlation coefficient (r) was used to evaluate the strength of the linear relationship between variables. For mouse data, comparisons between two or multiple groups were assessed using a Student`s t-test. All *P* values were 2-tailed and $P<0.05$ was considered statistically significant. Analyses were performed with GraphPad Prism software.

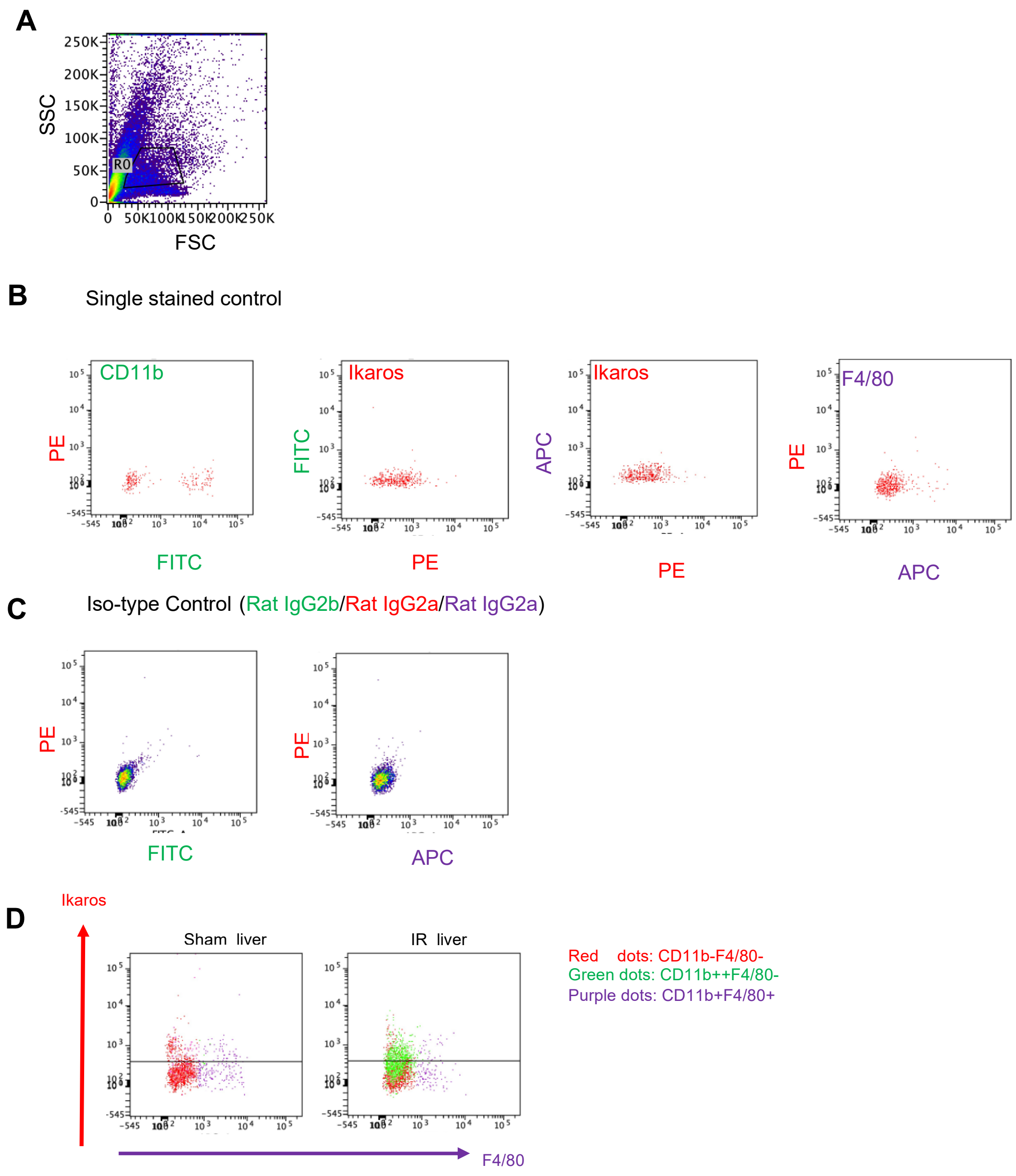

Fig.S1. Hepatic NPCs gating and control staining by FACS.

Liver NPCs were gated by forward scatter (FSC) and side scatter (SSC), as shown. **(B)** Single-stained controls were used for compensation and determination of cell-surface marker thresholds. **(C)** Iso-type control was used for validation of antibodies and determination of cell-surface marker thresholds. **(D)** Populations in Fig.2D are displayed based on F4/80 and Ikaros expression to dissect CD11b+F4/80+ populations.

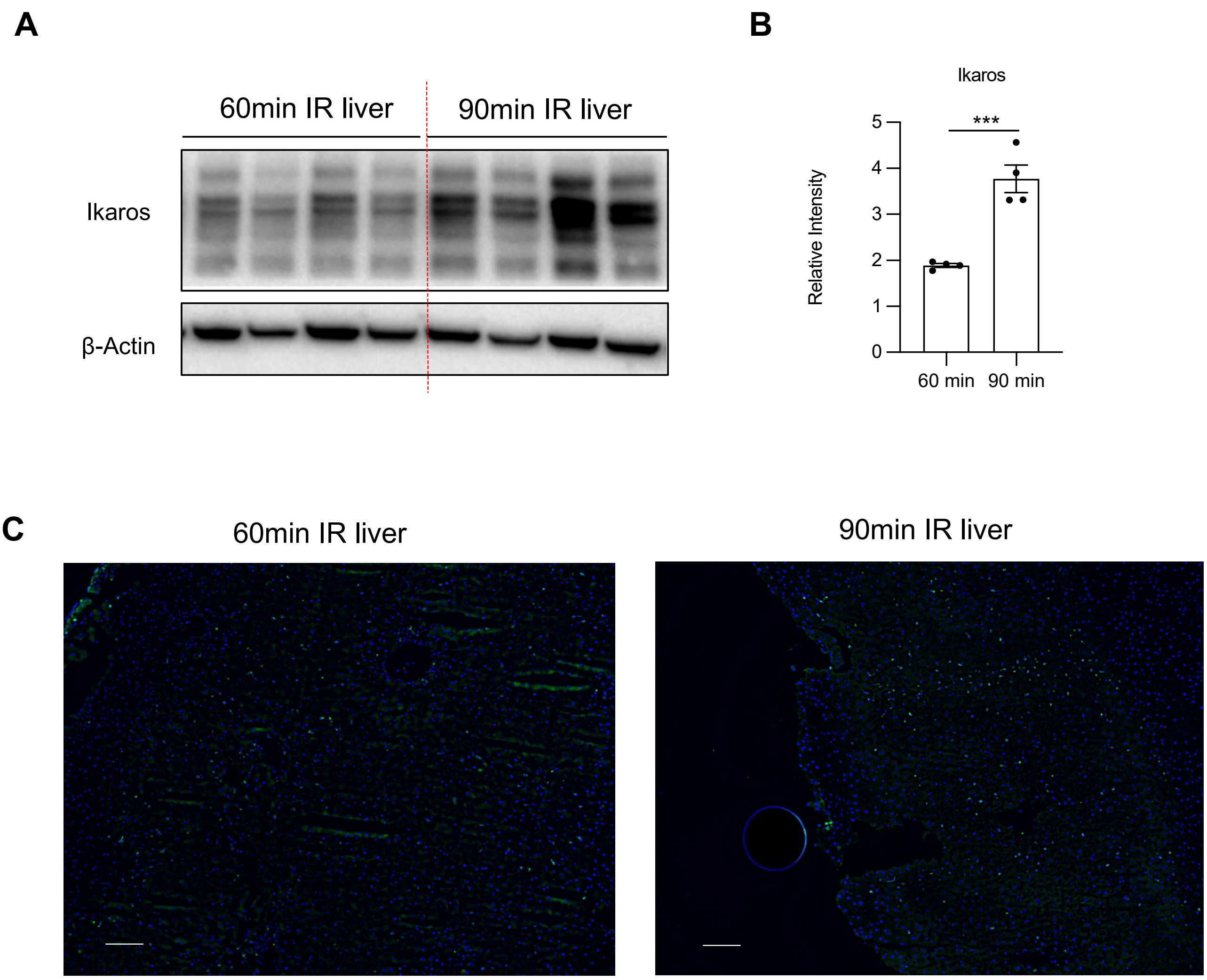

Fig.S2. Prolonged liver ischemia increases hepatic Ikaros expression and recruitment of CD11b+ cells.

WT mice were subjected to 60 min or 90 min warm ischemia, followed by 6 h reperfusion. **(A)** Western blot-assisted detection of Ikaros and β-actin as a loading control in IR-stressed livers (n=4/groups). **(B)** Relative intensity normalized by β-actin. **(C)** Representative immunofluorescence staining of CD11b (green) images of 60 min ischemia/reperfused liver *(Left panel)* and 90 min ischemia/reperfused liver (Right *panel*) (original magnification, ×100; scale bar, 100μm).

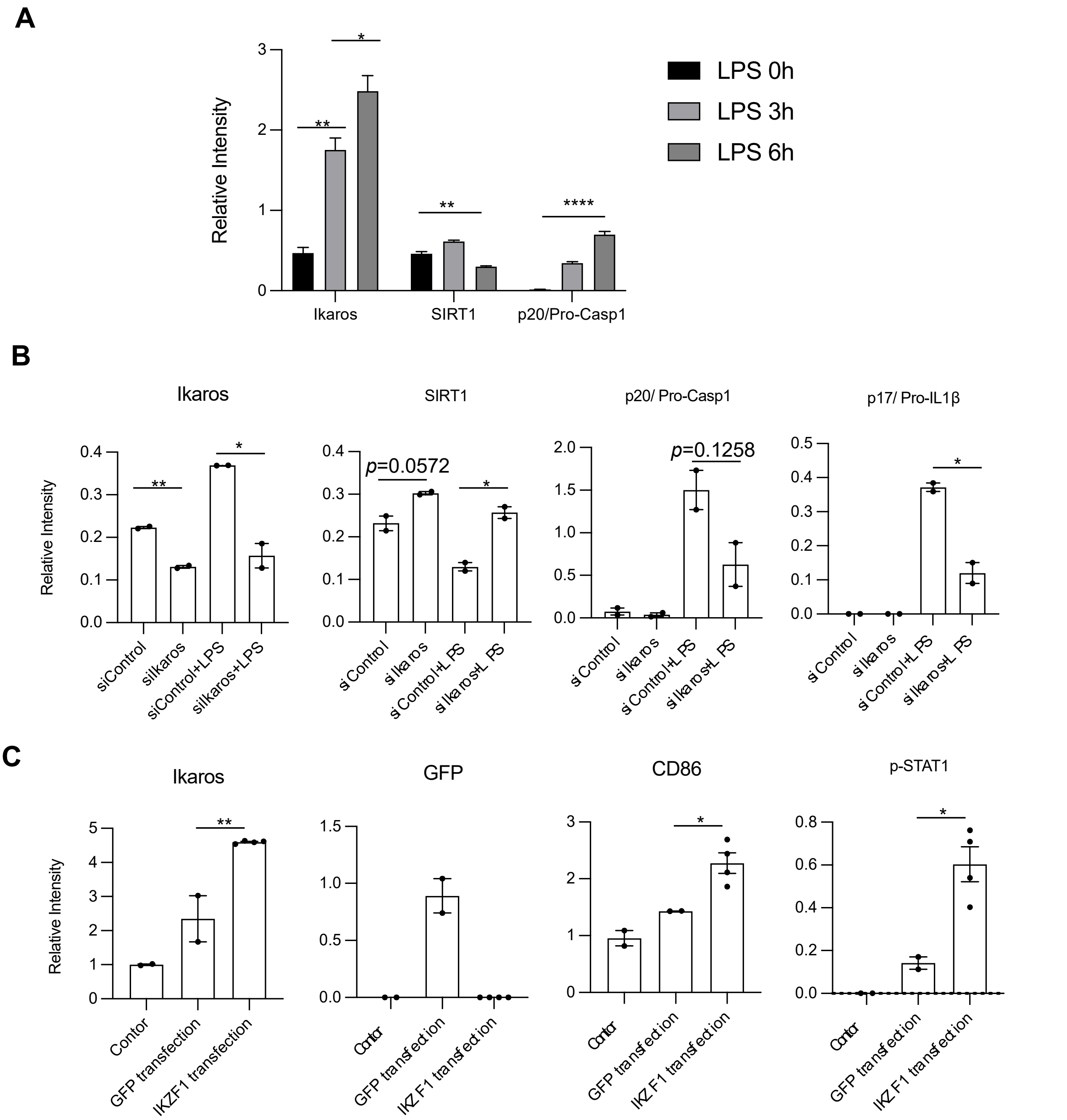

Fig.S3. Ikaros silencing suppresses macrophage inflammation/caspase-1 signaling.

(A) Relative intensity of Ikaros, SIRT1 and p20/Pro-Casp1 ratio with VCL normalization in Fig.3B. **(B)** Relative Intensity of Ikaros, SIRT1, p20/Pro-Casp1 ratio and p17/IL1βratio with VCL normalization in Fig.3D. **(C)** Relative intensity of Ikaros, GFP, CD86, p-Stat1 with VCL normalization in Fig.3G. Data shown are mean±SEM; n=3/group. *p<0.05; **p<0.01 by Student`s t-test.

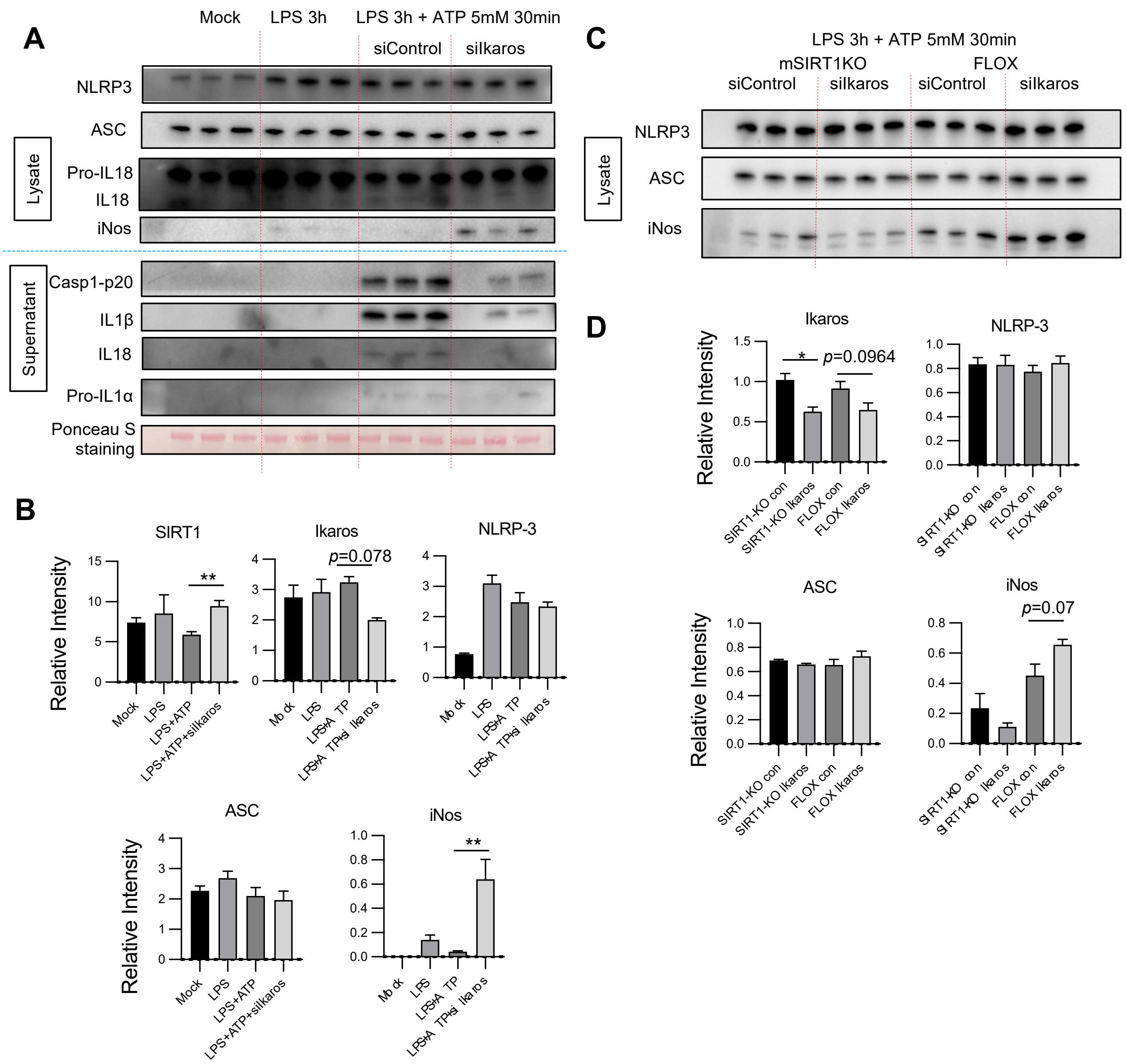

Fig.S4. Ikaros-SIRT1 axis in LPS/ATP-stimulated BMM are associated with iNos expression.

(A) Western blot-assisted detection of NLRP3, ASC, Pro-IL18, IL18 and iNos in Fig.4A (*Upper panel*). WB detection of Casp1-p20, IL1β, IL18 and Pro-IL1α in cell supernatants (*Lower panel*). **(B)** Relative intensity of the rest of Fig.4A blots or Fig.S5A are shown. **(C)** WB detection of NLRP3, ASC and iNos in Fig.4C. **(D)** Relative intensity of the rest of Fig.4C or Fig.S5D are shown. Data shown are mean±SEM; n=3/group. *p<0.05; **p<0.01 by Student`s t-test.

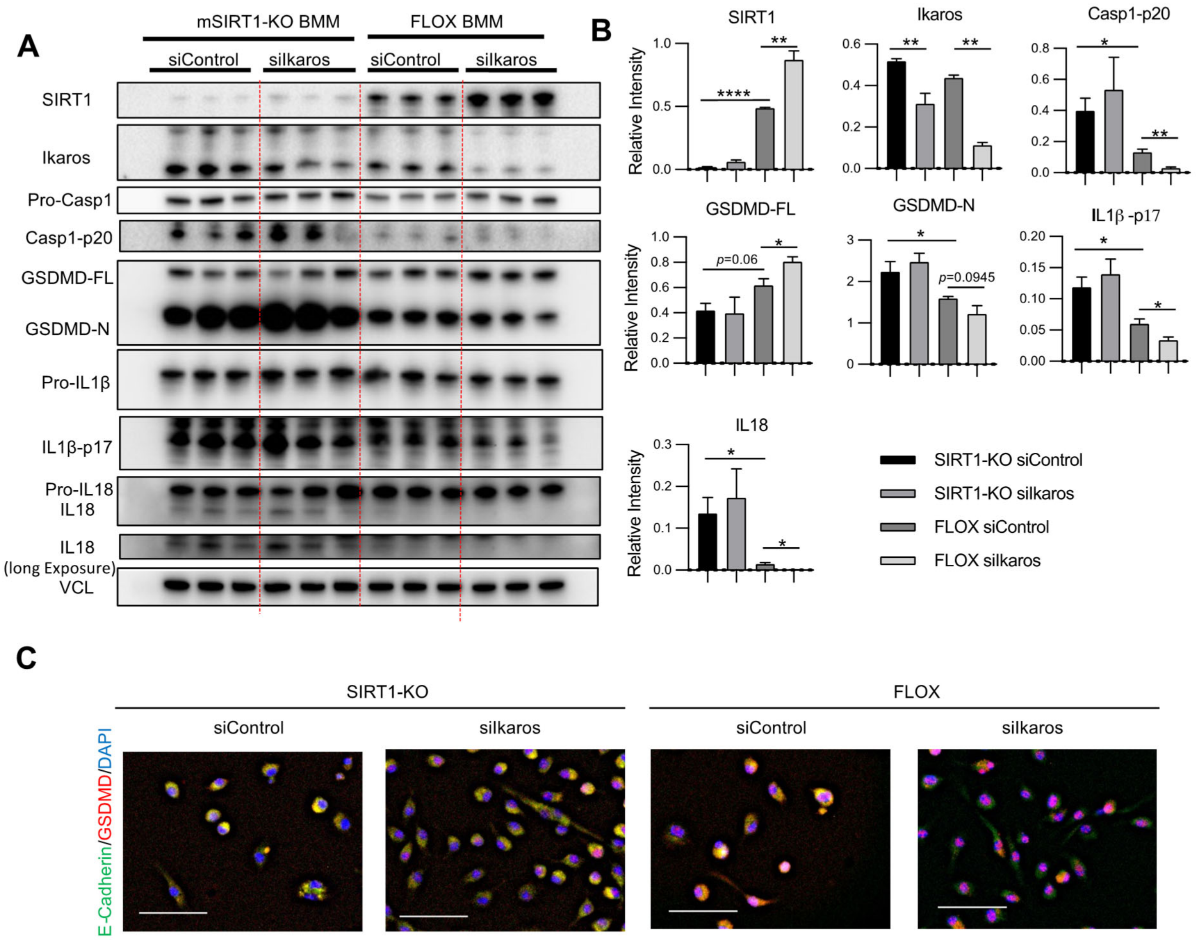

Fig.S5. Ikaros regulation of caspase-1-GSDMD processing in BMM is SIRT1-dependent.

BMM from FLOX and mSIRT1-KO mice were transfected with siIkaros or siControl RNAs and stimulated with LPS (100ng/ml, 6h). **(A)** Lysates from LPS-conditioned BMM were probed by Western blots to express SIRT1, Ikaros, Pro-Casp1, Casp1-p20, GSDMD-FL, GSDMD-N, Pro-IL1β,IL1β-p17, Pro-IL18, IL18, and VCL as a loading control. **(B)** Relative intensity ratios with VCL normalization. Data shown are mean±SEM; n=3/group. *p<0.05; **p<0.01; ****p<0.0001 by Student`s t-test. **(C)** Representative immunohistochemical detection of E-cadherin (green) and GSDMD (red) (original magnification, ×400; scale bar, 50μm).

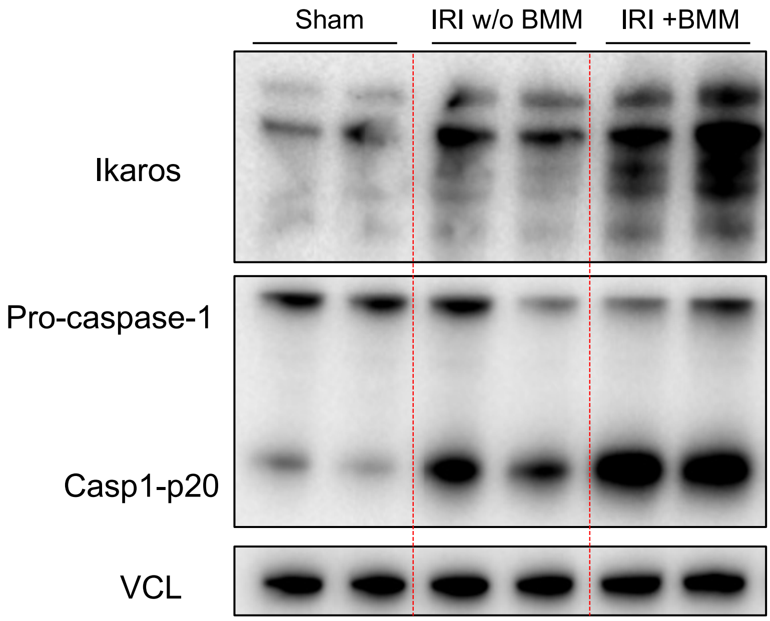

Fig.S6. Robust hepatic caspase-1 activation and Ikaros expression by macrophages recruited into IR-stressed liver.

CD11b-DTR mice untreated or reconstituted with BMM were subjected to 60 min warm ischemia and 6 h reperfusion. Representative Western-assisted detection of Ikaros, Casp1-p20 and VCL as a loading control. Experiments were repeated at least three times.

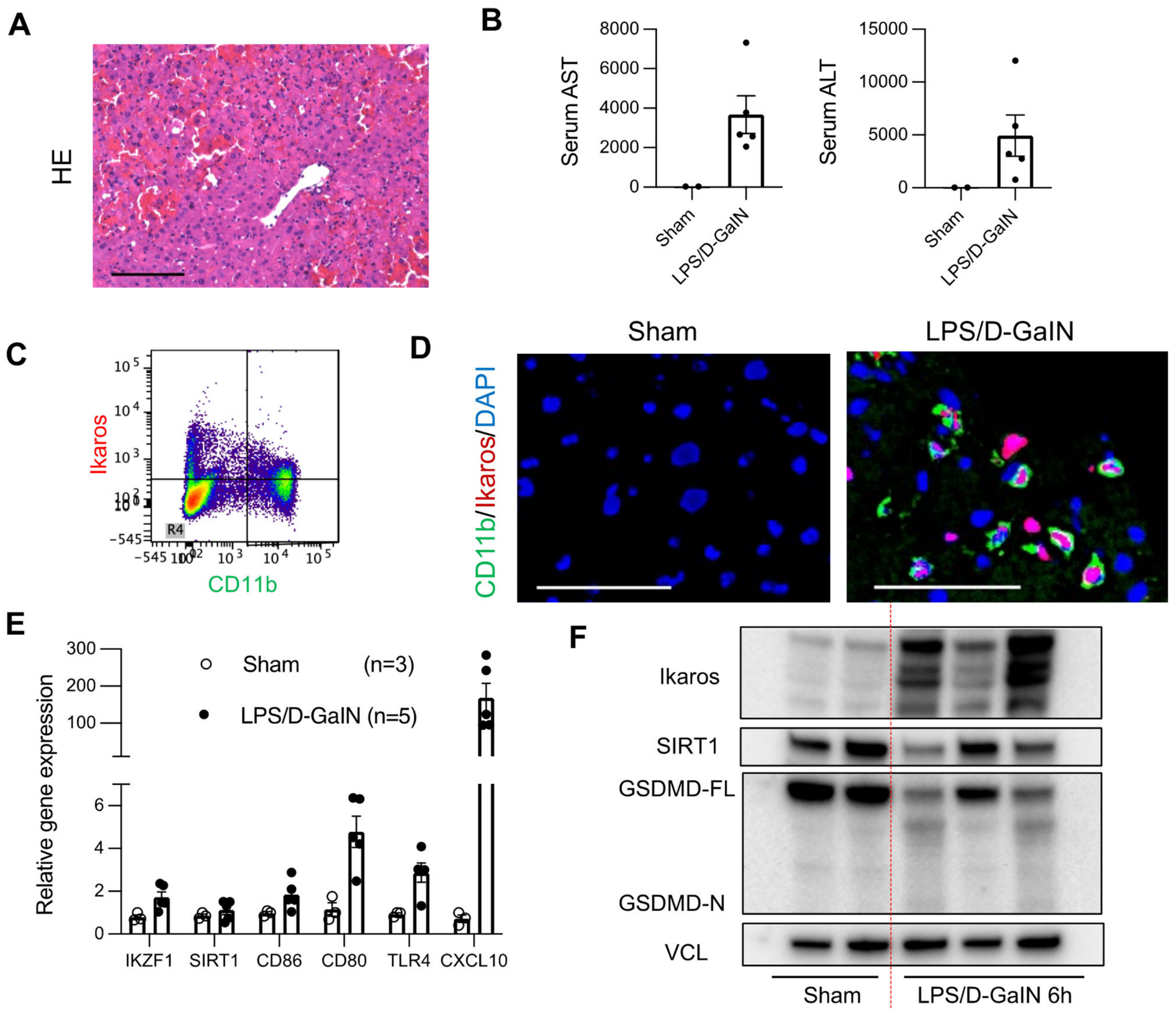

Fig.S7. Hepatic Ikaros expression by recruited macrophages in the acute liver hepatitis model.

WT mice were subjected to LPS/GalN treatment (as described in Methods). **(A)** Representative H&E (original magnification, ×200; scale bar 100μm). **(B)** Serum AST and ALT levels (n=2/Sham; n=5/LPS/GalN). **(C)** Representative FACS staining of CD11b and Ikaros in NPCs isolated from LPS/GalN injured-liver. **(D)** Representative immunofluorescence staining of CD11b (green) and Ikaros (red) (original magnification, ×400; scale bar 50μm). **(E)** qRT-PCR-assisted detection of mRNA coding for Ikaros, SIRT1, CD86, CD80, TLR4, and CXCL10. Data were normalized to HPRT expression (n=3/Sham; n=5/LPS/GalN). **(F)** Western-assisted detection of Ikaros, SIRT1, GSDMD and VCL as a loading control. Error bars shown are mean±SEM.

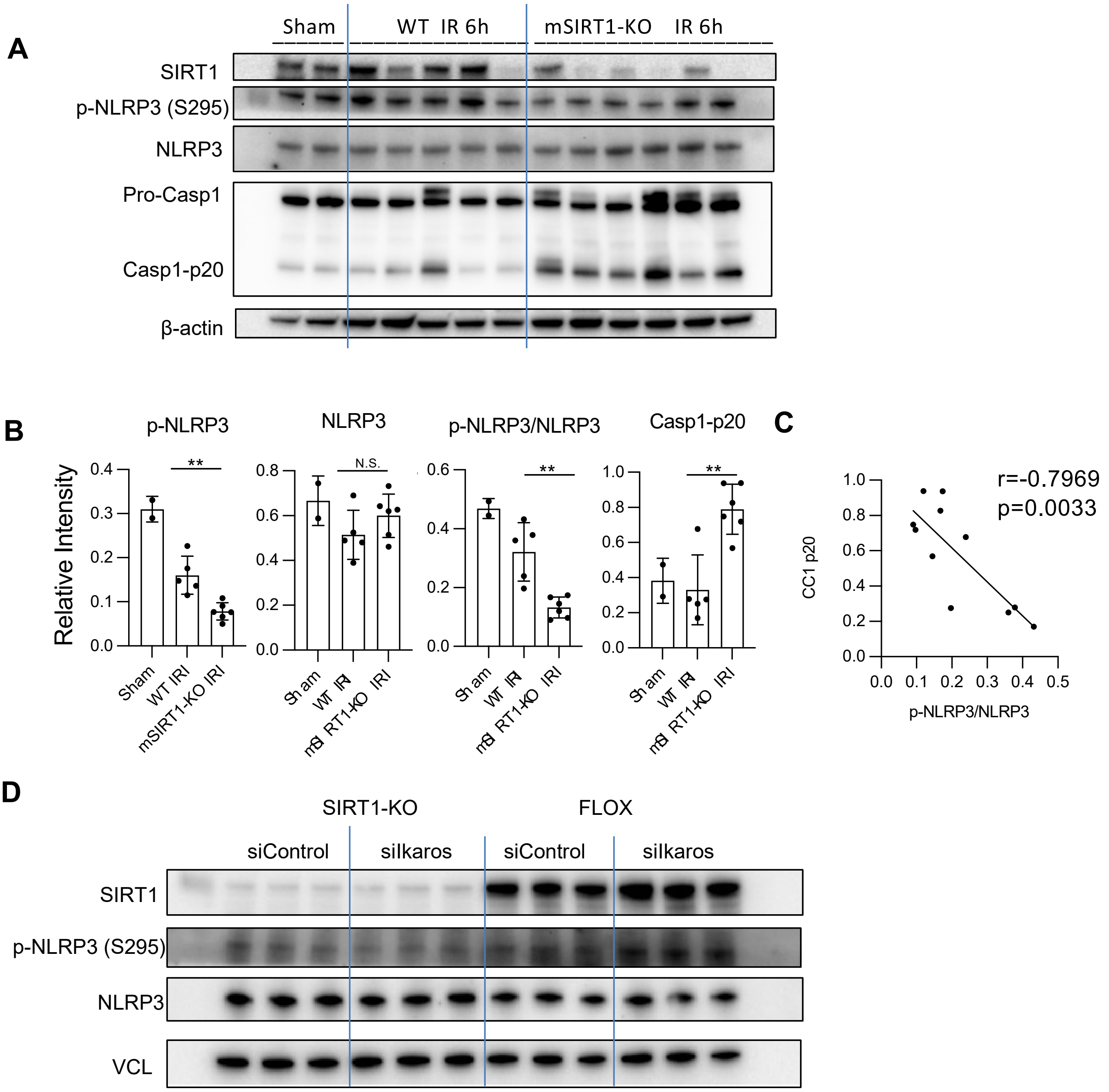

Fig.S8. SIRT1 expression associates with phosphorylated NLRP3 at Ser295.

FLOX control and mSIRT1-KO mice were subjected to warm IRI. **(A)** Lysates from FLOX-Sham, IR-liver and mSIRT1-KO IR-liver were probed by Western blots to express SIRT1, p-NLRP3, NLRP3, Pro-Casp1, Casp1-p20 and β-actin as a loading control. **(B)** Relative intensity ratios with VCL normalization. Data shown are mean±SEM; n=5-6/group. **p<0.01 by Student`s t-test. **(C)** The

association between p-NLRP3/NLRP3 ratio and CC1-p20 in FLOX or mSIRT1KO IR livers (n=11). Correlations were analyzed by nonparametric Speaman`s method. **(D)** Western blot-assisted detection of p-NLRP3 and NLRP3 in FLOX and mSIRT1-KO BMM.

Supplemental Table 1

Human liver transplant recipients` perioperative variables (55 clinical cases)

Variables	IKZF1 low (n=28)	IKZF1 high (n=27)	p value
Age (years)	55 (23-73)	58 (30-70)	0.528
Sex (M/F)	20 (71.4%) / 8 (28.5%)	17 (62.9%) / 10 (37.0%)	0.504
Race			0.270
White	15 (53.5%)	15 (55.6%)	
Hispanic	11 (39.2%)	9 (33.3%)	
Black	0	1 (3.7%)	
Asian	0	2 (7.4%)	
Others	2 (7.1%)	0	
Height (cm)	170.2 (149.9-193.0)	167.6 (149.9-190.5)	0.595
BMI (kg/m^2)	25.5 (14.5-47.5)	31.3 (17.1-44.3)	0.044
History			
Diabetes mellitus	7	12	0.130
Coronary artery disease	4	6	0.729
Hypertension	9	13	0.226
Disease etiology			0.709
HBV	3 (10.7%)	1 (3.7%)	
HCV	10 (35.7%)	10 (37.0%)	
EtOH	4 (14.2%)	3 (11.1%)	
Cryptogenic cirrhosis/NASH	4 (14.2%)	7 (25.9%)	
ALF	1 (3.6%)	0	
Others	6 (21.4%)	6 (22.2%)	
HCC (with/without)	11 (39.2%) / 17 (60.8%)	9 (33.3%) / 18 (66.7%)	0.646
ABO			0.531
Identical	27 (96.4%)	25 (92.6%)	
Compatible	1 (3.6%)	2 (7.4%)	
MELD score	34 (16-42)	31 (9-44)	0.098
Pretransplant AST (IU/L)	63 (26-1231)	56 (22-180)	0.496
Pretransplant ALT (IU/L)	31 (11-617)	36 (9-164)	0.704
Pretransplant T-Bil (mg/dL)	10.0 (0.3-59.1)	3.5 (0.5-49.2)	0.496
Preoperative Alb	3.7 (2.4-6.8)	3.4 (1.6-4.4)	0.054

Supplemental Table 2			
**Donors` perioperative variables (55 clinical cases)**			
**Variables**	**IKZF1 low (n=28)**	**IKZF1 high (n=27)**	**p value**
Age (years)	44 (18-67)	40 (13-66)	0.98
SEX (M/F)	11 (39.2%) / 17 (60.8%)	17 (63.0%) / 10 (37.0%)	0.079
BMI (Kg/m^2)	25.6 (13.4-42.6)	26.6 (19.6-32.6)	0.98
History			
Diabetes mellitus	4	3	0.724
Coronary artery disease	2	1	0.575
Hypertension	9	5	0.246
Cold Ischemia Time (min)	480 (150-1213)	435 (120-750)	0.197
Warm Ischemia Time (min)	50.5 (23-79)	54 (25-78)	0.400

Supplemental Table 3

Primer Sequences Used for Real-Time Quantitative PCR (mouse study)

Gene	Forward	Reverse
Mouse SIRT1	5`-ATCGGCTACCGAGACAAC-3`	5`-GTCACTAGAGCTGGCGTGT-3`
Mouse IL1β	5`-TGTAATGAAAGACGGCACACC-3`	5`-TCTTCTTTGGGTATTGCTTGG-3`
Mouse CXCL10	5`-GCTGCCGTCATTTTCTGC-3`	5`-TCTCACTGGCCCGTCATC-3`
Mouse TNFα	5`-CCTATGTCTCAGCCTCTCT-3`	5`-TTGGGAACTTCTCATCCCTT-3`
Mouse IKZF1	5`-ATGTCCCAAGTTTCAGGAAAGG-3`	5`-GCACGCCCATTCTCTTCATC-3`
Mouse iNos	5`-GTTCTCAGCCCAACAATACAAGA-3`	5`-GTGGACGGGTCGATGTCAC-3`
Mouse IL6	5`-GTACCATAGCTACCTGGAGT-3`	5`-GGAAATTGGGGTAGGAAGGA-3`
Mouse CD80	5`-ACCCCCAACATAACTGAGTCT-3`	5`-TTCCAACCAAGAGAAGCGAGG-3`
Mouse CD86	5`-GAGCTGGTAGTATTTTGGCAGG-3`	5`-GGCCCAGGTACTTGGCATT-3`
Mouse GAPDH	5`-AGGTCGGTGTGAACGGATTTG-3`	5`-AGGTCGGTGTGAACGGATTTG-3`
Mouse Ly6G	5`-TGGACTCTCACAGAAGCAAAG-3`	5`-GCAGAGGTCTTCCTTCCAACA-3`
Mouse IFNg	5`-CAGCAACAGCAAGGCGAAAAAGG-3`	5`-TTTCCGCTTCCTGAGGCTGGAT-3`
Mouse CD4	5`-AGGTGATGGGACCTACCTCTC-3`	5`-GGGGCCACCACTTGAACTAC-3`
Mouse β2m	5`-TGACCGGCTTGTATGCTATC-3`	5`-CACATGTCTCGATCCCAGTAG-3`

Supplemental Table 4

Primer Sequences Used for Real-Time Quantitative PCR (human study)

Gene	Forward	Reverse
Human IKZF1	5`-TTCCGTGATCCTTTTGAGTGC-3`	5`-CTCGCGTTATGTTATGTGCGACGA-3`
Human TLR4	5`-AGACCTGTCCCTGAACCCTAT-3`	5`-CGATGGACTTCTAAACCAGCCA-3`
Human Cathepsin G	5`-GAGTCAGACGGAATCGAAACG-3`	5`-CGGAGTGTATCTGTTCCCCTC-3`
Human CD80	5`-AAACTCGCATCTACTGGCAAA-3`	5`-GGTTCTTGTACTCGGGCCATA-3`
Human CD86	5`-CTGCTCATCTATACACGGTTACC-3`	5`-GGAAACGTCGTACAGTTCTGGATT-3`
Human CXCL10	5`-GTGGCATTCAAGGAGTACCTC-3`	5`-TGATGGCCTTCGATTCTGTG-3`
Human GAPDH	5`-GGAGCGAGATCCCTCCAAAAT-3`	5`-GGCTGTTGTCATACTTCTCATGG-3`

REFERENCES

1. Schug TT, Xu Q, Gao H, Peres-da-Silva A, Draper DW, Fessler MB*, et al.* Myeloid deletion of SIRT1 induces inflammatory signaling in response to environmental stress. Mol Cell Biol 2010;30:4712-4721.

2. Kusakabe J, Hata K, Miyauchi H, Tajima T, Wang Y, Tamaki I*, et al.* Complement-5 Inhibition Deters Progression of Fulminant Hepatitis to Acute Liver Failure in Murine Models. Cell Mol Gastroenterol Hepatol 2021.

3. Shohachi S, Luis H T-P, Francisco J R, Dolores C. Neutrophil infiltration as an important factor in liver ischemia and reperfusion injury. Modulating effects of FK506 and cyclosporine. Transplantation 1993;55:1265–1272.

4. Nakamura K, Zhang M, Kageyama S, Ke B, Fujii T, Sosa RA*, et al.* Macrophage heme oxygenase-1-SIRT1-p53 axis regulates sterile inflammation in liver ischemia-reperfusion injury. J Hepatol 2017;67:1232-1242.

5. Moradian H, Roch T, Lendlein A, Gossen M. mRNA Transfection-Induced Activation of Primary Human Monocytes and Macrophages: Dependence on Carrier System and Nucleotide Modification. Sci Rep 2020;10:4181.

6. **Nakamura K, Kageyama S**, Ke B, Fujii T, Sosa RA, Reed EF*, et al.* Sirtuin 1 attenuates inflammation and hepatocellular damage in liver transplant ischemia/Reperfusion: From mouse to human. Liver transplantation : official publication of the American Association for the Study of Liver Diseases and the International Liver Transplantation Society 2017;23:1282-1293.

7. Kageyama S, Kadono K, Hirao H, Nakamura K, Ito T, Gjertson DW*, et al.* Ischemia-Reperfusion Injury in Allogeneic Liver Transplantation: A Role of CD4 T Cells in Early Allograft Injury. Transplantation 2020.
