# Supplementary material for: Myeloid Ikaros–SIRT1 signaling axis regulates hepatic inflammation and pyroptosis in ischemia-stressed mouse and human liver
Source: J Hepatol. Author manuscript; Available in PMC 2022 Nov 28. (PMC9704689; doi:10.1016/j.jhep.2021.11.026)
Supplement: 2 [file NIHMS1852203-supplement-2.docx]

**Journal of Hepatology**

**CTAT methods**

Tables for a “Complete, Transparent, Accurate and Timely account” (CTAT) are now mandatory for all revised submissions. The aim is to enhance the reproducibility of methods.

- Only include the parts relevant to your study
- Refer to the CTAT in the main text as ‘Supplementary CTAT Table’
- Do not add subheadings
- Add as many rows as needed to include all information
- Only include one item per row

**If the CTAT form is not relevant to your study, please outline the reasons why:**

|  |
| --- |

- 1. **Antibodies**

| **Name** | **Citation** | **Supplier** | **Cat no.** | **Clone no.** |
| --- | --- | --- | --- | --- |
| Ikaros Rabbit mAb |  | Cell signaling | 14856 | D6N9Y |
| SIRT1 Rabbit mAb |  | Cell signaling | 9475 | D1D7 |
| iNos Rabbit mAb |  | Cell signaling | 13120 | D6B6S |
| Bcl-xl Rabbit mAb |  | Cell signaling | 2764 | 54H6 |
| GFP Rabbit mAb |  | Cell signaling | 2956 | D5.1 |
| p-AMPKα Rabbit mAb |  | Cell signaling | 2535 | 40H9 |
| AMPKα Ab |  | Cell signaling | 2532 |  |
| ASC Rabbit mAb |  | Cell signaling | 67824 | D2W8U |
| Vinculin Rabbit mAb |  | Cell signaling | 18799 | E1E9V |
| β-Actin Rabbit mAb |  | Cell signaling | 5125 | 13E5 |
| Mouse cleaved caspase-1 p20 Ab |  | Adipogen | Casper-1 |  |
| NLRP3 rat Ab |  | R and D | MAB7578 |  |
| Mouse IL1α Ab |  | R and D | AF-400-NA |  |
| p-NLRP3 Ab |  | Invitrogen | PA5-105071 |  |
| Human-caspase-1 Rabbit mAb |  | Abcam | ab207802 |  |
| Anti Gasdermin D Rabbit mAb |  | Abcam | ab209845 |  |
| IL1β Ab |  | Abcam | ab9722 |  |
| Mouse IL18 Ab |  | Abcam | ab71495 |  |
| E-Cadherin Ab |  | R and D | AF748 |  |
| rat anti-CD11b Ab |  | BD Biosciences | 556059 | M1/70 |
| rat anti-Ly6G Ab |  | BD Biosciences | 5502992 | RB6-8C5 |
| Ikaros PE Ab |  | Biolegend | 653304 | 2A9/Ikaros |
| CD11b FITC Ab |  | Biolegend | 101206 | M1/70 |
| F4/80 APC Ab |  | Biolegend | 123115 | BM6 |

- 1. **Cell lines**

| **Name** | **Citation** | **Supplier** | **Cat no.** | **Passage no.** | **Authentication test method** |
| --- | --- | --- | --- | --- | --- |
|  |  |  |  |  |  |

- 1. **Organisms**

| **Name** | **Citation** | **Supplier** | **Strain** | **Sex** | **Age** | **Overall n number** |
| --- | --- | --- | --- | --- | --- | --- |
|  |  |  |  |  |  |  |

- 1. **Sequence based reagents**

| **Name** | **Sequence** | **Supplier** |
| --- | --- | --- |
| Ikaros siRNA | sc-35641 | Santa Cruz |
| AMPKα1/2 siRNA | sc-45313 | Santa Cruz |

- 1. **Biological samples**

| **Description** | **Source** | **Identifier** |
| --- | --- | --- |
|  |  |  |

- 1. **Deposited data**

| **Name of repository** | **Identifier** | **Link** |
| --- | --- | --- |
|  |  |  |

- 1. **Software**

| **Software name** | **Manufacturer** | **Version** |
| --- | --- | --- |
| GraphPad Prism | Graphpad | 9.2.0 |

- 1. **Other (e.g. drugs, proteins, vectors etc.)**

| **Reagent** | **Cat no.** | **Supplier** |
| --- | --- | --- |
| Lipopolysaccharides | L5418-2ML | Sigma |
| ATP Solution | R0441 | Thermo Scientific |
| Compound C | 171261-1MG | EMD Millipore |

- 1. **Please provide the details of the corresponding methods author for the manuscript:**

| Kentaro Kadono, MD, PhD, Dumont-UCLA Liver Transplantation Center, Department of Surgery, David Geffen School of Medicine at UCLA ; 10833 Le Conte Ave, 77-120 CHS, Los Angeles, CA 90095. Email: [kkadono@mednet.ucla.edu](mailto:kkadono@mednet.ucla.edu). Phone: 1-310-825-5318 |
| --- |

**2.0 Please confirm for randomised controlled trials all versions of the clinical protocol are included in the submission. These will be published online as supplementary information.**

| **N/A** |
| --- |
